# Synthesis and Structure–Activity Relationship of 2,6-Disubstituted Thiosemicarbazone Derivatives of Pyridine as Potential Antituberculosis Agents

**DOI:** 10.3390/ma16010448

**Published:** 2023-01-03

**Authors:** Dagmara Ziembicka, Katarzyna Gobis, Małgorzata Szczesio, Andrzej Olczak, Ewa Augustynowicz-Kopeć, Agnieszka Głogowska, Izabela Korona-Głowniak, Krzysztof Bojanowski

**Affiliations:** 1 Department of Organic Chemistry, Faculty of Pharmacy, Medical University of Gdańsk, 107 Gen. Hallera Ave, 80-416 Gdansk, Poland; 2 Institute of General and Ecological Chemistry, Faculty of Chemistry, Lodz University of Technology, 116 Żeromskiego St, 90-924 Lodz, Poland; 3 Department of Microbiology, Institute of Tuberculosis and Pulmonary Diseases, 26 Płocka St, 01-138 Warsaw, Poland; 4 Department of Pharmaceutical Microbiology, Faculty of Pharmacy, Medical University of Lublin, 1 Chodźki St, 20-093 Lublin, Poland; 5 Sunny BioDiscovery Inc., 972 East Main St, Santa Paula, CA 93060, USA

**Keywords:** synthesis, pyridine, thiosemicarbazone, tuberculostatic activity, antimicrobial activity, cytotoxic activity, structure–activity relationship, X-ray, ADME

## Abstract

In this study, six new 2,6-disubstituted thiosemicarbazone derivatives of pyridine were synthesized (**4**–**9**), and their tuberculostatic activity was evaluated. All of them showed two- to eightfold higher activity (minimum inhibitory concentration (MIC) 0.5–4 µg/mL) against the resistant strain compared with the reference drug. Compounds **5** and **7**, which contained the most basic substituents—pyrrolidine and piperidine—in their structure, strongly inhibited the growth of the standard strain (MIC 2 µg/mL). Furthermore, the same derivatives exhibited activity comparable to that of the reference drugs against some types of Gram-positive bacteria (MIC 0.49 µg/mL) and showed no cytotoxicity (IC50 > 50 µg/mL) in HaCaT cells. The zwitterionic structure of each compound was determined using X-ray crystallography. Absorption, distribution, metabolism, and excretion analyses showed that all compounds are good drug candidates. Thus, compounds **5** and **7** were identified as leading structures for further research on antituberculosis drugs with extended effects.

## 1. Introduction

Tuberculosis (TB) is one of the first-identified contagious diseases and is caused by *Mycobacterium tuberculosis* [1,2]. Nevertheless, it remains a huge worldwide epidemiological problem, as evidenced by a recent report published by the World Health Organization [3]. The greatest obstacle in the implementation of TB control programs, such as the End TB strategy and TB-DOTS, is the emergence of drug-resistant, including multi-drug-resistant, *M. tuberculosis* (MDR-TB) strains [3,4]. The reasons for increased drug resistance include inadequate healthcare infrastructure, poor adherence to therapy, prescription of the wrong treatment, drug unavailability or using low-quality drugs, and reinfection, among others [5,6]. In 2021, 450,000 people developed MDR/RR-TB, and only 36% of confirmed cases were enrolled for treatment [3]. The treatment of MDR-TB, which is resistant to the two most potent drugs, isoniazid (INH) and rifampicin (RIF), involves using toxic and less effective second-line medications and is associated with a long treatment duration (up to 24 months) and a high cost of therapy, and it often fails to produce the desired outcomes [7,8]. The development of new drug regimens against MDR-TB had stagnated for decades until 2012 and 2014, when the EMA approved the use of bedaquiline and delamanid. However, the numerous side effects of these drugs make their application difficult. All these facts highlight the urgent need to discover subsequent drugs against TB [9,10,11].

**Figure 1:**
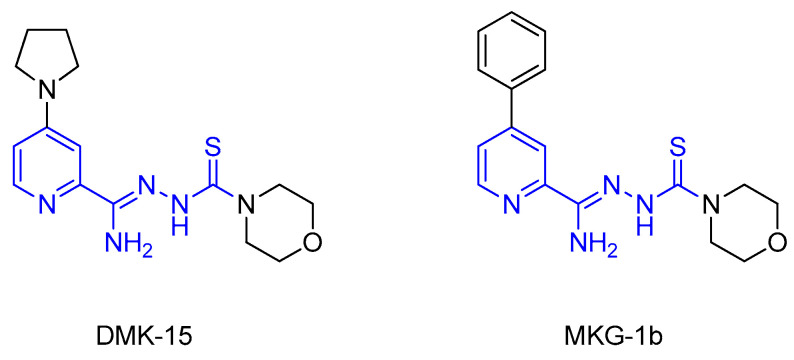
Structures of 2,4-disubstituted thiosemicarbazone derivatives of pyridine DMK-15 and MKG-1b.

In the recent literature, several series of compounds containing a pyridine ring [12,13,14] or a thiosemicarbazone fragment [15,16,17] have been reported with promising antituberculosis properties. These two elements were combined by our research team to create 2,4-disubstituted pyridine thiosemicarbazone derivatives. Leading structures, DMK-15 and MKG-1b (Figure 1), could be identified in each of the presented series [18,19]. DMK-15, with pyrrolidine on the 4-position of the pyridine ring and morpholine in the thiosemicarbazone chain, showed an excellent tuberculostatic potential (minimum inhibitory concentration (MIC) 0.4 µg/mL) with low cytotoxic activity on human dermal fibroblast cells (IC50 36.18 µg/mL). MKG-1b, with a phenyl group in the C-4 position and—similar to DMK-15—morpholine at the end of the side chain, showed high activity toward *Mycobacterium* strains (MIC 3.1 µg/mL), with a simultaneous low effect on other pathogenic microorganisms (MIC 0.98–500 µg/mL).

In these studies, we looked at how changing the position of the functional group’s connection to the aromatic ring from 4 to 6 affected the ability to inhibit mycobacteria. We created six 2,6-disubstituted thiosemicarbazone derivatives of pyridine for this purpose. The same or similar substituents were introduced into the structures, as in DMK-15 and MKG-1b. All compounds showed significant activity against the *M. tuberculosis*-resistant strain (MIC 0.5–4 µg/mL), whereas two of them also showed activity against the standard strain (MIC 2 µg/mL).

## 2. Materials and Methods

### 2.1. Chemistry

The initial compound (Apollo Scientific, Bredbury, UK) and all reagents and solvents (Sigma-Aldrich, Darmstadt, Germany) were of analytical grade. Thin-layer chromatography was performed on Merck silica gel 60F_254_ plates and visualized using UV light. The stationary phase in column chromatography was high-purity Merck silica gel (pore size 60 Å, 70–230 mesh). Elemental analyses (%C, H, N) of all synthesized compounds in the solid form were carried out using a PerkinElmer PE 2400 Series II CHNS analyzer (Perkin-Elmer, Shelton, CT, USA), the results of which were in agreement with the calculated values within the ±0.4% range. IR spectra were recorded as KBr pellets of the solids using a Satellite FT-IR spectrophotometer (Bruker, Madison, WI, USA). The ^1^H and ^13^C NMR spectra in DMSO-*d*_6_ were recorded using Varian Unity Plus (500 MHz) and Varian Gemini (200 MHz) instruments (Varian Medical Systems, Palo Alto, CA, USA). Melting points were determined using a Stuart SMP30 apparatus (Stone, Staffordshire, UK) and were uncorrected.

#### 2.1.1. General Procedure for the Synthesis of Nitriles **1**–**3**

First, 40 mmol of 6-chloropicolinonitrile and 48 mmol of an appropriate nucleophilic agent were dissolved in 25 mL of dioxane, and then, 6 mL of 1,8-diazabicyclo [5.4.0]undec-7-ene (DBU) was added. The mixture was refluxed for 1–12 h. After the evaporation of the solvent, ice was added. The precipitated products were filtered, dried, and recrystallized from a suitable solvent or purified by column chromatography.

##### 6-(Pyrrolidin-1-Yl)Picolinonitrile (**1**)

Starting from 6-chloropicolinonitrile (5.5 g) and pyrrolidine (4 mL), compound **1** was obtained as white crystals (6.3 g, 91%): m.p. 73–75 ℃ (methanol); IR (KBr): 3097, 3075 (υ C_Ar_-H), 2959, 2865 (υ C-H), 2230 (υ C≡N), 1616, 1595 (υ C=N), 1499, 1458 (υ C=C), 1247, 1225, 1207, 1185 (δ C-H), 793 (γ C-H) cm^−1^; ^1^H NMR (500 MHz, DMSO-*d*_6_): δ 1.92–1.95 (m, 4H, 2CH_2_), 4.58–3.84 (m, 4H, 2CH_2_), 6.75 (d, 1H, pyridine, J = 9 Hz), 7.08 (d, 1H, pyridine, J = 7 Hz), 7.62 (dd, 1H, pyridine, J_1_ = 8 Hz, J_2_ = 8 Hz) ppm; ^13^C NMR (125 MHz, DMSO-*d*_6_): δ 25.34 (2C), 46.85 (2C), 112.05, 116.69, 118.73, 130.93, 138.17, 157.15 ppm; Anal. Calcd for C_10_H_11_N_3_ (173.21): C, 69.34; H, 6.40; N, 24.26; Found: C, 69.38; H, 6.17; N, 24.52.

##### 6-(Piperidin-1-Yl)Picolinonitrile (**2**)

Starting from 6-chloropicolinonitrile (5.5 g) and piperidine (4.7 mL), compound **2** was obtained as slightly yellowish liquid (6.9 g, 92%): m.p.—(AcOEt:CHCl_3_ 1:5); IR (KBr): 3019 (υ C_Ar_-H), 2939, 2856 (υ C-H), 2234 (υ C≡N), 1595 (υ C=N), 1487, 1446 (υ C=C), 1254, 1215 (δ C-H), 771 (γ C-H) cm^−1^; ^1^H NMR (500 MHz, DMSO-*d*_6_): δ 1.51–1.54 (m, 4H, 2CH_2_), 1.0–1.61 (m, 2H, CH_2_), 2.50–2.51 (m, 4H, 2CH_2_), 7.11 (d, 1H, pyridine, J = 7 Hz), 7.15 (d, 1H, pyridine, J = 9 Hz), 7.64 (dd, 1H, pyridine, J_1_ = 7 Hz, J_2_ = 7 Hz) ppm; ^13^C NMR (125 MHz, DMSO-*d*_6_): δ 24.54, 25.36 (2C), 45.60 (2C), 112.19, 117.41, 118.56, 130.75, 138.86, 159.04 ppm. The results of the elemental analyses are consistent with a description in the literature [20,21].

##### 6-Phenoxypicolinonitrile (**3**)

Starting from 6-chloropicolinonitrile (5.5 g) and phenol (4.5 g), compound **3** was obtained as white crystals (7.5 g, 91%): m.p. 72–74 ℃ (AcOEt:CHCl_3_ 1:5); IR (KBr): 3080, 3041 (υ C_Ar_-H), 2239 (υ C≡N), 1586, 1567 (υ C=N), 1491, 1433 (υ C=C), 1258 (δ C-H), 1210 (υ C-O), 802, 767, 704, 686 (γ C-H) cm^−1^; ^1^H NMR (500 MHz, DMSO-*d*_6_): δ 7.20 (d, 2H, Ph, J = 8 Hz), 7.28 (t, 1H, Ph, J = 8 Hz), 7.41 (d, 1H, pyridine, J = 9 Hz), 7.47 (t, 2H, Ph, J = 7 Hz), 7.79 (d, 1H, pyridine, J = 7 Hz), 8.08 (t, 1H, pyridine, J = 8 Hz) ppm; ^13^C NMR (125 MHz, DMSO-*d*_6_): δ 117.41, 117.45, 121.79 (2C), 125.06, 125.83, 129.87, 130.38 (2C), 142.28, 153.23, 163.65 ppm; Anal. Calcd for C_12_H_8_N_2_O (196.20): C, 73.46; H, 4.11; N, 14.28; Found: C, 73.43; H, 4.27; N, 14.18.

#### 2.1.2. General Procedure for the Synthesis of Thiosemicarbazones **4**–**9**

##### Method A (**4**, **6**)

A solution of the nitrile (2 mmol) in methanol (10 mL) was treated with DBU (2.7 mmol, 0.4 mL) and heated to reflux for 4 h. Then, 2 mmol of carbothiohydrazide was added, and the mixture was refluxed for another 1–1.5 h. The reaction mixture was poured onto ice and acidified with acetic acid, which resulted in precipitation. The precipitated products were filtered, dried, and purified by column chromatography.

##### Method B (**7**)

A solution of the nitrile (2 mmol) in methanol (10 mL) was treated with DBU (2.7 mmol, 0.4 mL) and heated to reflux for 4 h. Then, 2 mmol of carbothiohydrazide was added, and the mixture was refluxed for another 2.5 h. The reaction mixture was poured onto ice and acidified with acetic acid, resulting in the formation of an oily suspension, which was extracted with chloroform (3 × 20 mL). The combined organic layers were dried over anhydrous MgSO_4_. The drying agent was filtered, and the solvent evaporated. After triple washing with diethyl ether, a precipitate was formed. The precipitated product was dried and recrystallized from a suitable solvent.

##### Method C (**5**, **8**, **9**)

A solution of the nitrile (2 mmol) in methanol (10 mL) was treated with DBU (2.7 mmol, 0.4 mL) and heated to reflux for 4 h. Then, 2 mmol of carbothiohydrazide was added, and the mixture was refluxed for another 0.5–1 h. Then, the reaction mixture was cooled, resulting in the formation of a precipitate. The precipitated products were filtered, dried, and recrystallized from a suitable solvent.

##### *N*’-(Morpholine-4-Carbonothioyl)-6-(Pyrrolidin-1-Yl)Picolinohydrazonamide (**4**)

Starting from 6-(pyrrolidin-1-yl)picolinonitrile (0.411 g) and morpholine-4-carbothiohydrazide (0.322 g), the title compound **4** was obtained as yellow crystals (0.576 g, 86%): m.p. 180–182 °C (AcOEt:CHCl_3_ 1:1); IR (KBr): 3422, 3286, 3119 (υ N-H), 2957, 2851 (υ C-H), 1663 (υ C=N), 1608 (δ N-H), 1504, 1424 (υ C=C), 1350, 1309, 1261, 1221 (υ C-N), 1114 (υ C-O), 1021 (δ C-H), 889, 794 (γ C-H) cm^−1^; ^1^H NMR (500 MHz, DMSO-*d_6_*): δ 1.96–1.99 (m, 4H, 2CH_2_), 3.40–3.60 (m, 8H, 4CH_2_), 3.80–3.83 (m, 4H, 2CH_2_), 6.69 (d, 1H, pyridine, J = 9 Hz), 7.31 (d, 1H, pyridine, J = 7 Hz), 7.51 (br. s, 1H, NH_2_ + D_2_O exchangeable), 7.68 (t, 1H, pyridine, J = 8 Hz), 8.43 (br. s, 1H, NH_2_ + D_2_O exchangeable), 12.60 (br. s, 1H, NH + D_2_O exchangeable) ppm; ^13^C NMR (125 MHz, DMSO-*d*_6_): δ 25.41 (2C), 46.83 (2C), 47.24 (2C), 66.73 (2C), 108.77, 110.85, 138.64, 142.23, 145.75, 156.16, 179.11 ppm; Anal. Calcd for C_15_H_22_N_6_OS (334.44): C, 53.87; H, 6.63; N, 25.13; Found: C, 53.74; H, 6.65; N, 24.79.

##### 6-(Pyrrolidin-1-Yl)-*N*’-(Pyrrolidine-1-Carbonothioyl)Picolinohydrazonamide (**5**)

Starting from 6-(pyrrolidin-1-yl)picolinonitrile (0.411 g) and pyrrolidine-1-carbothiohydrazide (0.290 g), the title compound **5** was obtained as yellow crystals (0.425 g, 61%): m.p. 200–201 °C (anhydrous ethanol); IR (KBr): 3407, 3243 (υ N-H), 3061 (υ C_Ar_-H), 2969, 2837 (υ C-H), 1664 (υ C=N), 1599 (δ N-H), 1497, 1460 (υ C=C), 1161, 1101 (υ C-N), 1015, 917 (δ C-H), 788 (γ C-H) cm^−1^; ^1^H NMR (500 MHz, DMSO-*d_6_*): δ 1.81–1.96 (m, 8H, 4CH_2_), 3.47–3.56 (m, 8H, 4CH_2_), 6.67 (d, 1H, pyridine, J = 9 Hz), 7.28 (d, 1H, pyridine, J = 8 Hz), 7.51 (br. s, 1H, NH_2_ + D_2_O exchangeable), 7.67 (t, 1H, pyridine, J = 8 Hz), 8.20 (br. s, 1H, NH_2_ + D_2_O exchangeable), 12.59 (br. s, 1H, NH + D_2_O exchangeable) ppm; ^13^C NMR (125 MHz, DMSO-*d*_6_): δ 25.25 (2C), 25.41 (2C), 46.82 (2C), 48.60 (2C), 108.53, 110.54, 138.62, 142.38, 144.26, 156.15, 176.41 ppm; ppm; Anal. Calcd for C_15_H_22_N_6_S (318.44): C, 56.58; H, 6.96; N, 26.39; Found: C, 56.51; H, 7.03; N, 26.15.

##### *N*’-(Morpholine-4-Carbonothioyl)-6-(Piperidin-1-Yl)Picolinohydrazonamide (**6**)

Starting from 6-(piperidin-1-yl)picolinonitrile (0.374 g) and morpholine-4-carbothiohydrazide (0.322 g), the title compound **6** was obtained as yellow crystals (0.510 g, 73%): m.p. 177–178 °C (AcOEt:CHCl_3_ 1:1); IR (KBr): 3280, 3221, 3134 (υ N-H), 3082 (υ C_Ar_-H), 2932, 2848 (υ C-H), 1671 (υ C=N), 1607 (δ N-H), 1496, 1461 (υ C=C), 1382, 1350, 1252, 1223 (υ C-N), 1114 (υ C-O), 1020 (δ C-H), 889, 791 (γ C-H) cm^−1^; ^1^H NMR (500 MHz, DMSO-*d_6_*): δ 1.53–1.63 (m, 6H, 3CH_2_), 3.56–3.67 (m, 8H, 4CH_2_), 3.80–3.81 (m, 4H, 2CH_2_), 7.08 (d, 1H, pyridine, J = 9 Hz), 7.33 (d, 1H, pyridine, J = 8 Hz), 7.52 (br. s, 1H, NH_2_ + D_2_O exchangeable), 7.70 (t, 1H, pyridine, J = 8 Hz), 8.42 (br. s, 1H, NH_2_ + D_2_O exchangeable), 12.62 (br. s, 1H, NH + D_2_O exchangeable) ppm; ^13^C NMR (125 MHz, DMSO-*d*_6_): δ 24.76, 25.44 (2C), 45.94 (2C), 47.24 (2C), 66.72 (2C), 109.58, 111.09, 139.32, 142.10, 145.53, 158.07, 179.93 ppm; Anal. Calcd for C_16_H_24_N_6_OS (348.47): C, 55.15; H, 6.94; N, 24.12; Found: C, 55.35; H, 6.76; N, 23.89.

##### 6-(Piperidin-1-Yl)-*N*’-(Pyrrolidine-1-Carbonothioyl)Picolinohydrazonamide (**7**)

Starting from 6-(piperidin-1-yl)picolinonitrile (0.374 g) and pyrrolidine-1-carbothiohydrazide (0.290 g), the title compound **7** was obtained as yellow crystals (0.379 g, 57%): m.p. 166–168 °C (toluene); IR (KBr): 3433, 3295 (υ N-H), 3054 (υ C_Ar_-H), 2931, 2853 (υ C-H), 1671 (υ C=N), 1603 (δ N-H), 1533, 1461 (υ C=C), 1361, 1337, 1274, 1236 (υ C-N), 1026 (δ C-H), 914, 787 (γ C-H) cm^−1^; ^1^H NMR (500 MHz, DMSO-*d_6_*): δ 1.53–1.63 (m, 6H, 3CH_2_), 1.82–1.83 (m, 4H, 2CH_2_), 3.55–3.66 (m, 4H, 2CH_2_), 7.05 (d, 1H, pyridine, J = 9 Hz), 7.30 (d, 1H, pyridine, J = 7 Hz), 7.51 (br. s, 1H, NH_2_ + D_2_O exchangeable), 7.69 (t, 1H, pyridine, J = 8 Hz), 8.20 (br. s, 1H, NH_2_ + D_2_O exchangeable), 12.62 (br. s, 1H, NH + D_2_O exchangeable) ppm; ^13^C NMR (125 MHz, DMSO-*d*_6_): δ 24.77, 25.24 (2C), 25.46 (2C), 45.95 (2C), 48.60 (2C), 109.35, 110.77, 139.29. 142.23, 144.04, 158.07, 176.51 ppm; Anal. Calcd for C_16_H_24_N_6_S (332.47): C, 57.80; H, 7.28; N, 25.28; Found: C, 57.49; H, 7.20; N, 24.88.

##### *N*’-(Morpholine-4-Carbonothioyl)-6-Phenoxypicolinohydrazonamide (**8**)

Starting from 6-phenoxypicolinonitrile (0.393 g) and morpholine-4-carbothiohydrazide (0.322 g), the title compound **8** was obtained as yellow crystals (0.152 g, 21%): m.p. 131–134 °C (ethanol); IR (KBr): 3453, 3373, 3173 (υ N-H), 3049 (υ C_Ar_-H), 2970, 2851 (υ C-H), 1683 (υ C=N), 1588, 1574 (δ N-H), 1458, 1441 (υ C=C), 1341, 1253, 1227, 1223 (υ C-N), 1108 (υ C-O), 1023 (δ C-H), 908, 803 (γ C-H) cm^−1^; ^1^H NMR (500 MHz, DMSO-*d_6_*): δ 3.53–3.55 (m, 4H, 2CH_2_), 3.77–3.79 (m, 4H, 2CH_2_), 6.46 (br. s, 1H, NH), 7.19–7.28 (m, 4H, pyridine + 3Ph), 7.42 (t, 2H, pyridine, J = 8 Hz), 7.70 (br. s, 1H, NH), 7.87 (d, 1H, pyridine, J = 8 Hz), 8.09 (t, 1H, pyridine, J = 8 Hz), 8.45 (br. s, 1H, NH) ppm; ^13^C NMR (125 MHz, DMSO-*d*_6_): δ 47.16 (2C), 66.69 (2C), 115.09, 116.86, 121.55 (2C), 125.52, 130.45 (2C), 142.48, 142.57, 144.64, 153.29, 162.80, 179.66 ppm; Anal. Calcd for C_17_H_19_N_5_O_2_S (357.43): C, 57.13; H, 5.36; N, 19.59; Found: C, 57.09; H, 5.21; N, 19.36.

##### 6-Phenoxy-*N*’-(Pyrrolidine-1-Carbonothioyl)Picolinohydrazonamide (**9**)

Starting from 6-phenoxypicolinonitrile (0.393 g) and pyrrolidine-1-carbothiohydrazide (0.290 g), the title compound **9** was obtained as yellow crystals (0.338 g, 41%): m.p. 170–173 °C (ethanol); IR (KBr): 3417, 3212 (υ N-H), 3038 (υ C_Ar_-H), 2968, 2852 (υ C-H), 1666 (υ C=N), 1591, 1574 (δ N-H), 1445, 1424 (υ C=C), 1342, 1273, 1237 (υ C-N), 1107 (υ C-O), 1026 (δ C-H), 797, 703 (γ C-H) cm^−1^; ^1^H NMR (500 MHz, DMSO-*d_6_*): δ 1.81–1.84 (m, 4H, 2CH_2_), 3.52–3.53 (m, 4H, 2CH_2_), 3.73–3.74 (m, 4H, 2CH_2_), 6.36 (s, 2H, NH_2_), 7.14–7.28 (m, 3H, pyridine + 3Ph), 7.43–7.47 (t, 2H, Ph, J = 7 Hz), 7.75 (d, 1H, pyridine, J = 8 Hz), 8.08 (t, 1H, pyridine, J = 8 Hz), 9.23 (s, 1H, NH) ppm; ^13^C NMR (125 MHz, DMSO-*d*_6_): δ 25.26 (2C), 48.59, 52.86, 115.55, 116.58, 121.43 (2C), 125.43, 130.40 (2C), 142.42, 142.72, 143.23, 153.37, 162.75, 176.98 ppm; Anal. Calcd for C_17_H_19_N_5_OS (341.43): C, 59.80; H, 5.61; N, 20.51; Found: C, 59.47; H, 5.21; N, 20.27.

### 2.2. Tuberculostatic Activity Assay

Compounds **4**–**9** were examined in vitro for their tuberculostatic activity toward two *M. tuberculosis* strains: the standard H_37_Rv strain and a “wild-type” strain isolated from TB patients, namely Spec. 210, which is resistant to the clinically used antituberculosis drugs INH, RIF, ethambutol, and p-aminosalicylic acid. Investigations were performed in 96-well microtiter plates by twofold serial microdilution using Middlebrook 7H9 Broth medium (Beckton Dickinson) containing 10% of OADC (Beckton Dickinson). The inoculum was prepared from fresh LJ culture in Middlebrook 7H9 Broth medium with OADC, adjusted to a no. 0.5 McFarland tube, and diluted 1:100. The stock solution of the tested agent was prepared in DMSO. For each test compound, stock solutions were diluted in Middlebrook 7H9 Broth medium with OADC to achieve a fourfold value of the final highest concentration to be tested. Compounds were diluted serially in sterile 96-well microtiter plates using 100 µL Middlebrook 7H9 Broth medium with OADC. Concentrations of the tested agents ranged from 512 to 0.0625 µg/mL. A growth control step containing no antibiotic and a sterile control step without inoculation were also performed on each plate. The plates were incubated at 37 °C for 21 days. After the incubation period, 30 µL of Alamar Blue solution was added to each well, and the plates were reincubated for 24 h. Growth was indicated by a color change from blue to pink, and the lowest concentration of a compound that prevented the color change was noted as its MIC [22,23]. INH was used as the reference drug for comparison. Each experiment was performed in triplicate.

### 2.3. In Vitro Antibacterial Activity Assay

The antibacterial and antifungal activities of compounds **4**–**9** were screened by the microdilution broth method using Mueller–Hinton broth for the growth of bacteria and Mueller–Hinton broth with 2% glucose for the growth of fungi. The antimicrobial assays were carried out following a previous study [24]. The MICs of the aforementioned derivatives were evaluated for the panel of reference microorganisms from the ATCC (American Type Culture Collection), including Gram-positive bacteria (*Staphylococcus aureus* ATCC 25923, *Staphylococcus epidermidis* ATCC 12228, *Micrococcus luteus* ATCC 10240, *Bacillus subtilis* ATCC 6633, and *Bacillus cereus* ATCC 10876), Gram-negative bacteria (*Escherichia coli* ATCC 25922, *Proteus mirabilis* ATCC 12453, *Klebsiella pneumoniae* ATCC 13883, and *Pseudomonas aeruginosa* ATCC 9027), and fungi (*Candida albicans* ATCC 102231 and *Candida parapsilosis* ATCC 22019) (LGC Standards, Teddington, Middlesex, UK). Vancomycin (VAN), ciprofloxacin (CIP), and fluconazole (FCZ) were used as standard drugs for comparison. Each experiment was performed in triplicate.

### 2.4. Cytotoxic Activity Assay

Compounds **4**–**9** were stored at room temperature and were dissolved in DMSO at 20 mg/mL on the day of the experiment. Further dilutions were made in the cell growth medium. HaCaT cells, an immortalized human keratinocyte line (AddexBio, San Diego, CA, USA; gender: male; age: 62 years; tissue: skin), were plated at 30,000 cells/well in a 96-well plate without phenol red DMEM supplemented with 10% fetal bovine serum and penicillin–streptomycin–amphotericin (HiMedia (India)/Sigma-Aldrich, Saint Louis, MO, USA). Other test compounds were diluted in water to obtain 20× concentrated stock solutions, which were then added in triplicate to cell cultures at 20× dilution. After 3 days of incubation, cell viability was assayed using the MTT method, which measures cellular metabolism by quantifying the activity of dehydrogenases, such as succinate dehydrogenase, involved in the respiratory electron transport chain in mitochondria (which is proportional to cell viability) [25]. The total amount of proteins (proportional to cell numbers) was then assayed using the sulforhodamine B method [26]. The colorimetric signal was acquired using a Molecular Devices microplate reader MAX190 and SoftMax3.1.2PRO software. Statistical significance was evaluated using two-tailed student’s *t*-tests. Deviations ≥20% compared with water control with *p* values below 0.05 were considered statistically significant.

### 2.5. X-ray Study

Single crystals of compounds suitable for X-ray diffraction were obtained by slow evaporation of the solvents at room temperature, from methanol–DMF (1:1 *v*/*v*) for **5**–**9**, and from ]isopropanol–DMF–water (1:1:1 *v*/*v*) for **4**. Diffraction measurements were carried out on an XtaLAB Synergy diffractometer, Dualflex, (Rigaku Oxford Diffraction, PL, London, UK), with a Pilatus 300 K detector at low temperature (100 K) and with CuKα (1.54184 A) radiation. Diffraction data were processed using CrysAlis PRO (Rigaku Oxford Diffraction. CrysAlis PRO; Rigaku Oxford Diffraction Ltd.: Yarnton, Oxfordshire, England). Crystal structure solution and refinement were carried out using SHELX [27,28]. All H atoms (except those involved in hydrogen bonds) were geometrically optimized and allowed as riding atoms, with distances appropriate for a temperature of 100 K and with Uiso(H) = 1.2 Ueq(C, N). Methyl H atoms were refined with Uiso(H) = 1.5 Ueq(C).

In **5**, the highest maximum in the difference Fourier map was ~0.6 e/A-3, which is twice as large as the deepest hole on the map. This maximum was located near the sulfur atom, and its presence can probably be attributable to the anharmonicity of the motion of the sulfur atom since the anharmonic refinement in olex2 [29] reduces this maximum to 0.3 e/A-3. Similarly, for **7**, the highest maximum on the differential electron density map was twice as large as the deepest hole on the map. This disproportion can be reduced by the anharmonic refinement of sulfur atoms. In **8**, a disorder of the morpholine ring was detected, which was divided into two parts with the occupancy factors 0.59 and 0.41 and refined isotropically. In **6**, a large disorder was detected in the piperidine ring of molecule B. The ring was divided into two parts and refined with occupancy factors 0.83 and 0.17. Unfortunately, an additional disorder of water molecules near the threefold axis was much more difficult to refine; therefore, the SQUEEZE procedure was used. The number of electrons found in the solvent-accessible volume was 20, which gave two water molecules in the unit cell. In **4**, a significant disorder around the threefold axis was detected; therefore, the SQUEEZE procedure was used again. The number of electrons found in the solvent-accessible volume was 112, which can be interpreted as three isopropanol molecules and one water molecule in a unit cell. CCDC 2216086, 2216085, 2216084, 2216082, 2208427, and 2,208,428 contain the supplementary crystallographic data for this study. The data were provided free of charge by The Cambridge Crystallographic Data Centre via www.ccdc.cam.ac.uk/structures (accessed on 31 October 2022).

### 2.6. Absorption, Distribution, Metabolism, and Excretion (ADME)

The pharmacokinetic properties, drug-likeness, and absorption of the synthesized compounds were analyzed. An ADME analysis was carried out using the SwissADME service (Swiss Institute of Bioinformatics 2021 [30] and BOILED-Egg—To Predict Gastrointestinal Absorption and Brain Penetration of Molecules) [31].

## 3. Results and Discussion

### 3.1. Chemistry

**Figure 2:**
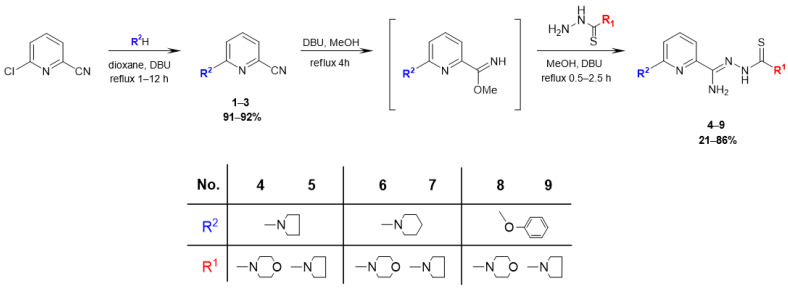
Synthesis of 2,6-disubstituted thiosemicarbazone derivatives of pyridine **4**–**9**.

The synthesis route used in this study is outlined in Figure 2. The initial compound 6-chloropicolinonitrile was refluxed for 1–12 h with a nucleophilic agent (pyrrolidine, piperidine, phenol) and then DBU was added to dioxane, yielding 6-substituted picolinonitriles **1**–**3** (yield 91–92%). Subsequently, the nitrile groups were converted into methyl imidate groups. These reactions were carried out at reflux for 4 h in the presence of methanol and a catalytic amount of DBU. To obtain final products **4**–**9** (yield 21–86%), each of the methyliminoesters, which were in the solution form prepared in situ, was condensed with morpholine-4-carbothiohydrazide and pyrrolidine-1-carbothiohydrazide by heating at boiling temperature for 0.5–2.5 h. Carbothiohydrazides were obtained in the reaction between methyl hydrazinecarbodithioate and cyclic amines in water or alcohol, following the modified method described by Klayman et al. [32]. All the newly synthesized compounds were characterized using the following methods: IR, ^1^H NMR, and ^13^C NMR spectra, elemental analysis. The results of the spectral analysis were in accordance with the assigned structures. The ^1^H NMR and ^13^C NMR spectra of all compounds are provided in Supplementary Materials in Figures S1–S18.

### 3.2. Tuberculostatic Activity

In vitro tuberculostatic activity against *M. tuberculosis* strains H_37_Rv and Spec. 210 is presented in Table 1 and is expressed in MICs, which is the lowest concentration needed to inhibit the growth of the tested microorganisms in relation to the control with no tested compound. INH was used as the reference drug. The MIC values of the tested compounds were in the range of 0.5–16 μg/mL, which indicates good-to-moderate antituberculosis activity. Compounds **5** and **7** showed the highest ability to inhibit the growth of bacterial cells. The structure of these compounds contained pyrrolidine and piperidine, respectively, located at the C6 position of the pyridine ring, and pyrrolidine in the thiosemicarbazone chain. Compared with the MIC values of INH (0.125 and 8 µg/mL), compounds **5** and **7** showed values that were 16 times higher (MIC 2 µg/mL) for the standard strain; however, more importantly, compound **7** showed a values that was 16 times lower (MIC 0.5 µg/mL), and compound **5** showed an value that was 8 times (MIC 1 µg/mL) for the resistant strain. The presence of a phenoxy moiety in **8** and **9** (with lower basicity and a higher molecular weight than piperidine and pyrrolidine) at the 6-position on the pyridine ring and the presence of morpholine in **4**, **6**, and **8** (with lower lipophilicity and higher electron density than pyrrolidine) on the side chain decreased the antituberculosis potency for both the standard strain (MIC 8–16 µg/mL) and the resistant strain (MIC 1–4 µg/mL). These derivatives showed activity that was two- to eightfold higher against the resistant strain as compared with the reference drug INH. Interestingly, for all compounds, lower inhibitory titers were observed for the resistant strain compared with that of the standard strain.

The *M. tuberculosis* H_37_Rv strain is used as a reference strain in various microbiological tests, including drug resistance tests, and in the search for new antimycobacterial drugs. Whole-genome sequencing of the reference strain and clinical strains revealed considerable genetic diversity between *M. tuberculosis* clinical isolates. The absence of some genes has also been proven in the genome of *M. tuberculosis* H_37_Rv in comparison with the genomes of clinical strains, e.g., genes encoding adenylate cyclase (MT1360), glycosyl transferase (MT1800), oxidoreductase (MT1801), and others. This genetic diversity observed in studies comparing the H_37_Rv genome with the genomes of clinical isolates contributes to the differences in pathogenicity and virulence of the strains and can explain the phenotypic variation of the strains. In addition, this fact may explain the higher MIC of the reference strain compared with the clinical strains [33,34,35,36].

### 3.3. Antimicrobial Activity

In vitro antimicrobial activity is presented in Table 2, which is expressed in MICs. CIP, VAN, and FCZ were used as reference drugs. Compounds **4**–**7** and **9** showed higher bacteriostatic activity against all types of Gram-positive bacteria—except for *B. cereus* ATCC 10,876 for **5**—than against tuberculostatic activity. For example, for compound **7**, the MIC value for all *Staphylococcus* strains was 0.49 μg/mL, whereas for the *M. tuberculosis* standard strain, it was 2 μg/mL. Moreover, their growth inhibition potential was equal to or higher than those of the reference drugs CIP and VAN, respectively. Compound **8** showed moderate or weak (MIC 31.3–250 μg/mL) to no activity (MIC > 1000 μg/mL) depending on the strain. None of the tested derivatives showed inhibitory effects on the growth of Gram-negative bacteria strains. A similar relationship was observed for fungi, except for *C. albicans* ATCC 102,231 for **4**, **5**, and **7,** and *C. parapsilosis* ATCC 22,019 for **7**, with good (MIC 7.8–15.6 μg/mL) or moderate activity (MIC 250 μg/mL).

### 3.4. Cytotoxic Activity

Cytotoxic activity in HaCaT cell lines determined using the two methods is outlined in Table 3 and is expressed as the half-maximal inhibitory concentration (IC50), which is defined as a measure of the strength of a substance to inhibit a specific biological or biochemical function. Compounds **4**, **5**, and **7** showed no cytotoxicity against noncancer cells. Compounds **6** and **8** inhibited cell metabolic activity while maintaining cell viability, whereas compound **9** showed the same while losing cell viability. The selectivity index (SI) was calculated by comparing IC50 values and determined using the sulforhodamine B method, for noncancerous epithelial HaCaT cells against MIC values for *M. tuberculosis* standard strains. The nontoxicity of compounds **4**–**8** was indicated by SI values > 1.0. For compounds **5** and **7**, which showed the highest tuberculostatic activity, the MIC value was more than 25 times lower, as compared with the concentration causing a cytotoxic effect against noncancer cells.

### 3.5. X-ray Study

**Figure 3:**
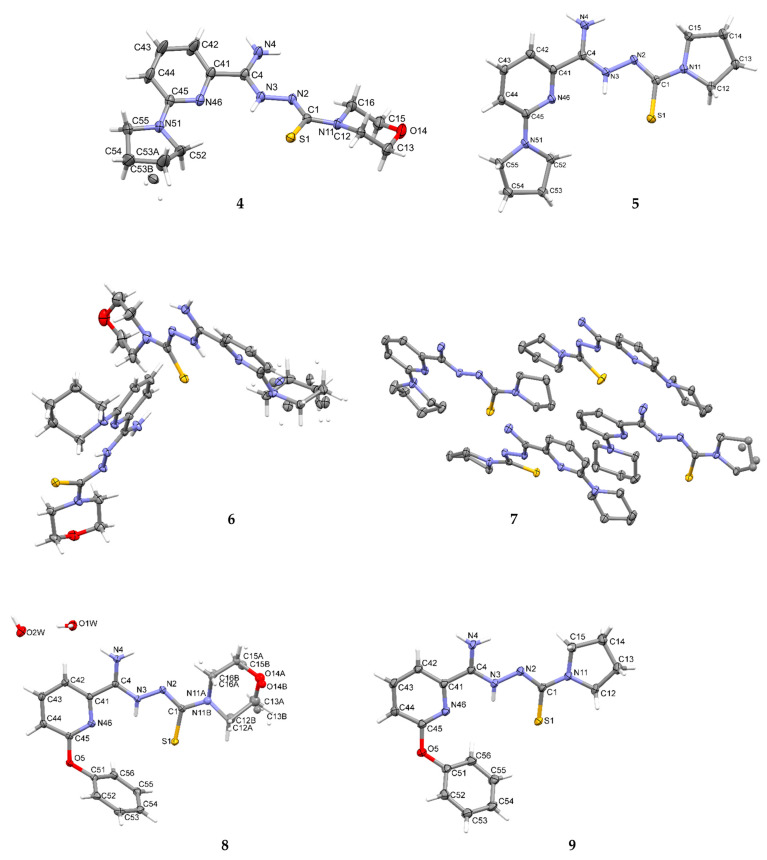
The molecular structures of compounds **4**–**9** showing the atom-labeling schemes. Displacement ellipsoids are drawn at the 50% probability level, and H atoms are shown as small spheres of arbitrary radii. Drawings were prepared with Mercury software.

**Figure 4:**
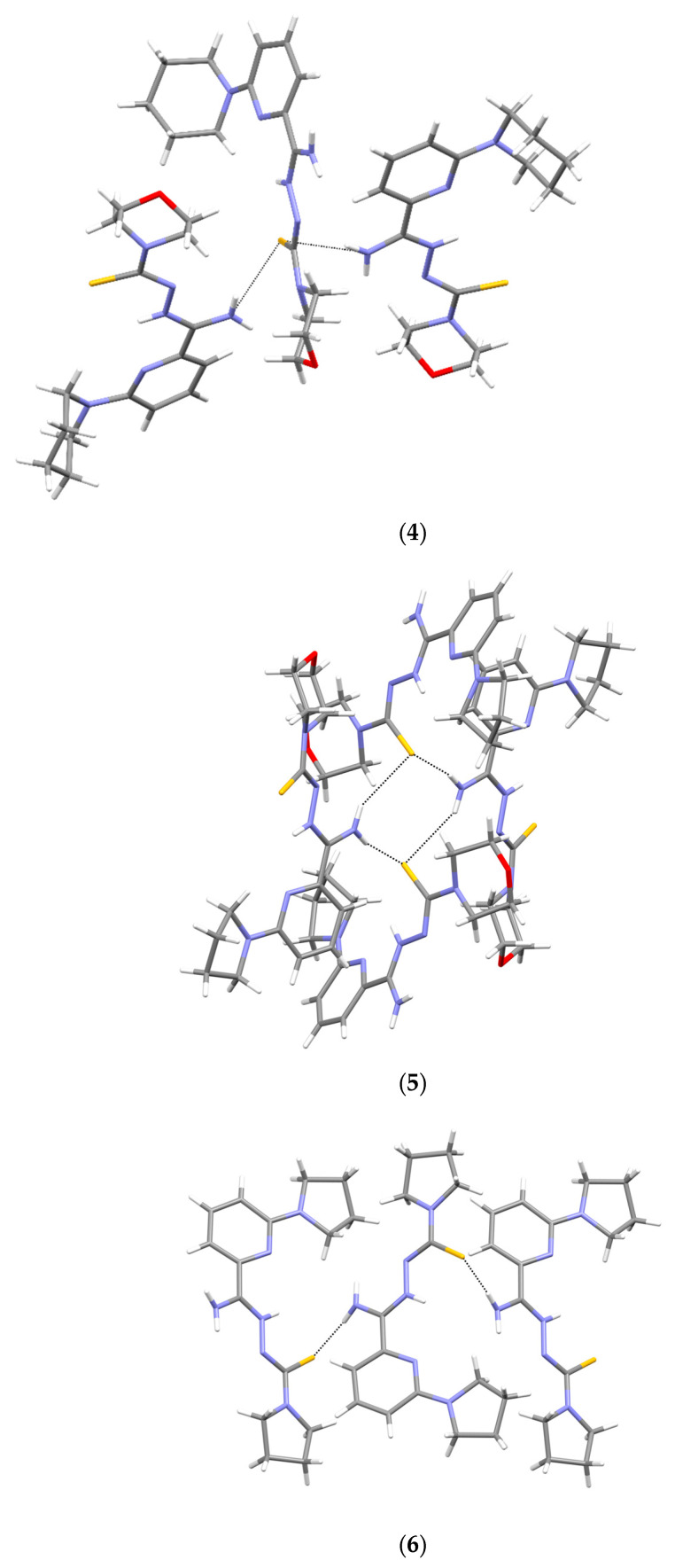

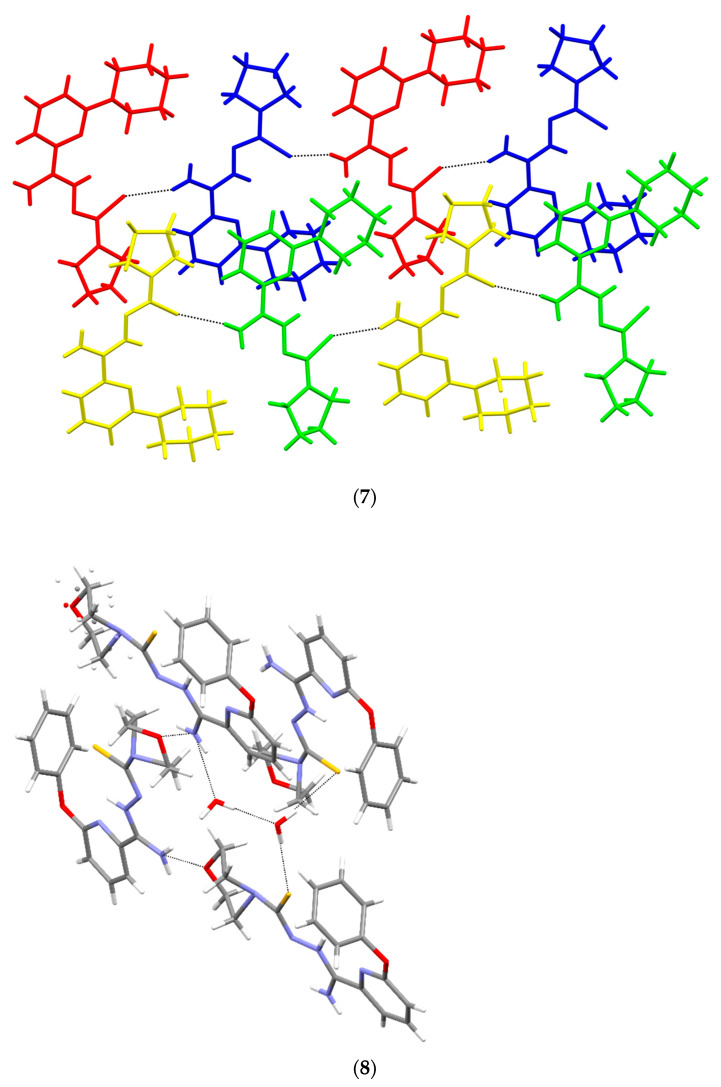

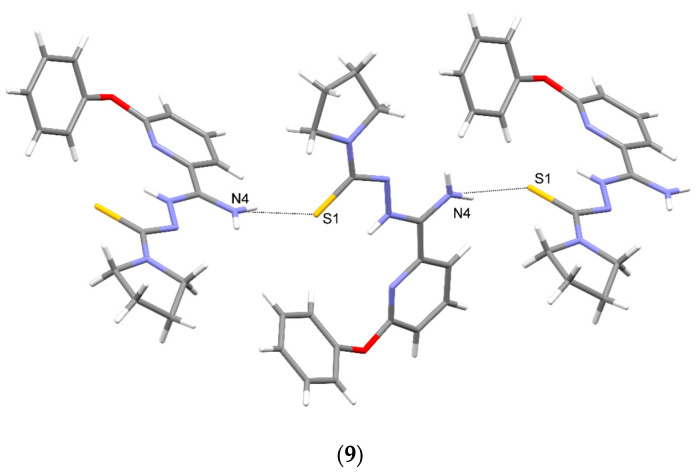
Intermolecular hydrogen bonds in **4**–**9**.

Crystallographic data are presented in Table 4 and Table 5. All tested compounds in the crystal state assumed the zwitterionic form (Figure 3). Four of the structures, namely **4**, **6**–**8**, showed static disorder. For trigonal structures, namely compounds **4** and **6**, the disorder was so significant that the SQUEEZE procedure had to be applied. Three structures, namely **4**, **6**, and **8**, were solvates, but the positions of the water molecules were well-defined only in compound **8**. Strong hydrogen bonds occurring in the studied compounds are presented in Table 6, Table 7, Table 8, Table 9, Table 10 and Table 11 and in Figure 4. Compounds **5**, **7**, and **9** showed the same hydrogen bond system. A chain hydrogen bond of the N–H···S type was formed (Figure 4, example for **9**). A chain bifurcated hydrogen bond of the N–H···S···H–N type was formed in compound **6** (Figure 4). The most complex system of hydrogen bonds was observed in compound **8**, in which water molecules formed hydrogen bonds with each other and with the NH_2_ group and the sulfur atom (Figure 4). Four molecules in the asymmetric unit were identified in compound **7**. Due to hydrogen bonding, molecules A and B and molecules C and D formed chains along the [110] crystallographic direction. These chains, in turn, established planes parallel to the plane $\left(\bar{1}11\right)$ in the crystal.

### 3.6. ADME Analysis

**Figure 5:**
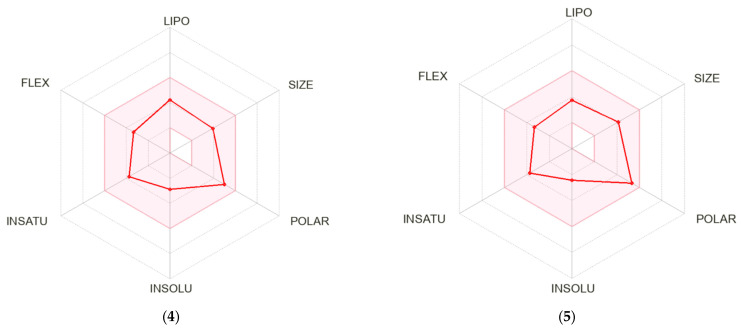

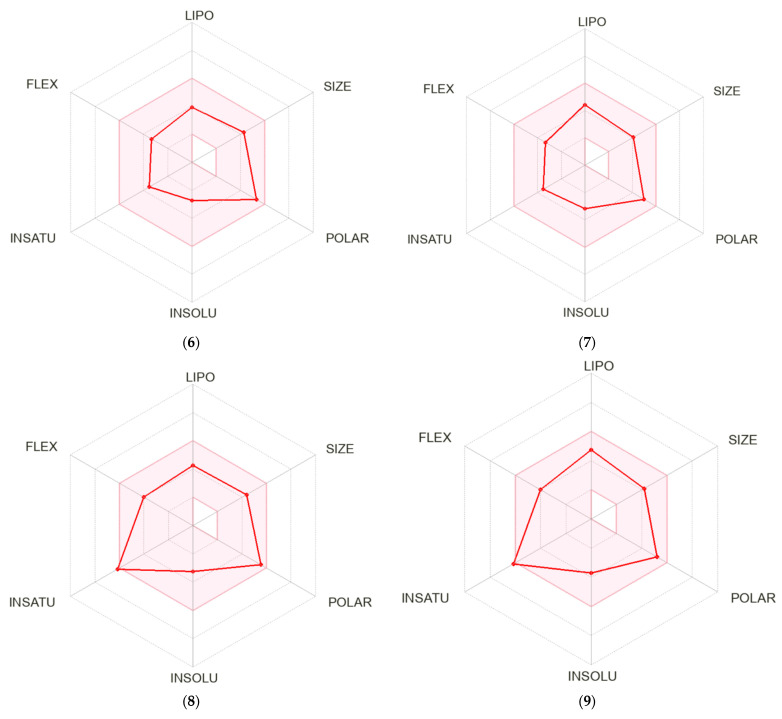
Bioavailability radar for **4**–**9**.

**Figure 6:**
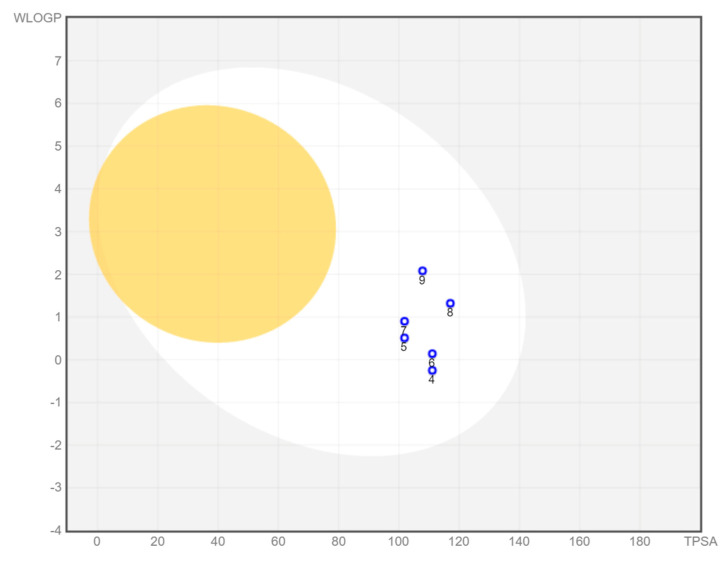
BOILED-Egg diagram for all compounds (lipophilicity (WLOGP) and polarity (tPSA), human intestinal absorption (white area), and blood–brain barrier permeation (yellow area).

A bioavailability radar was created for each compound studied (Figure 5). With respect to drug-likeness, the compounds were found to have a good bioavailability score (0.55). All compounds were found to conform to the rules of Lipinski [37], Ghose [38], Egan [39], Veber [40], and Muegge [41]. Thus, all compounds are good drug candidates. The logKp value for the tested compounds ranged from −7.61 cm/s to −6.39 cm/s, and the more negative the logKp, the less skin-permeant the molecule. In the BOILED-Egg diagram (Figure 6), all compounds showed absorption in the gastrointestinal tract, which may make them effective drugs. None of the compounds permeated the blood–brain barrier. All compounds were actively effluxed by P-glycoprotein, represented as (PGP+), which is indicated by the blue color of the indicator of the compound.

## 4. Conclusions

The synthesized compounds **4**–**9** had a pyrrolidine, piperidine, and phenoxy moiety at the 6-position on the pyridine ring and a thiosemicarbazone chain terminated with morpholine or pyrrolidine at the 2-position. The compounds were synthesized in three steps: substitution of a halogen atom of the initial material, their conversion to methyliminoesters, and condensation with carbothiohydrazides. The spatial structure of the compounds revealed that they crystallize in the zwitterionic form. Zwitterionic species were observed, probably due to the formation of an intramolecular hydrogen bond. All compounds showed a good bioavailability score and absorption in the gastrointestinal tract, and none of them permeated the blood–brain barrier, which makes them good drug candidates. Moreover, tuberculostatic, antimicrobial, and cytotoxic studies confirmed the drug potential of these compounds. The whole group of compounds is of particular interest because of their strong activity against the *M. tuberculosis*-resistant strain. Compounds containing pyrrolidine (**5**, **7**, **9**) show increased antibacterial activity. All these compounds exhibit the same hydrogen bonding systems. Compound **9** has a lower level of antituberculosis activity; this may be due to the introduced large phenoxy group. Compounds **5** and **7** can be identified as leading structures which additionally showed an effect on the standard *M. tuberculosis* strain and on other microorganisms and were nontoxic on nontumor cells.

## Figures and Tables

**Table 1:** In vitro tuberculostatic activity of compounds **4**–**9**.

Compound	MIC [µg/mL]	
	H_37_Rv	Spec. 210
**4**	16	4
**5**	2	1
**6**	8	1
**7**	2	0.5
**8**	8	4
**9**	8	4
INH	0.125	8

**Table 2:** In vitro antimicrobial activity against Gram-positive bacteria, Gram-negative bacteria, and fungi of compounds **4**–**9**.

Chemicals Microorganism	4	5	6	7	8	9	CIP	VAN	FCZ
	MIC [µg/mL]								
Gram-positive bacteria									
*S. aureus* ATCC 25923	3.9	0.49	1.95	0.49	250	0.98	0.49	0.98	-
*S. epidermidis* ATCC 12228	1.95	0.49	0.98	0.49	31.3	0.49	0.49	0.98	-
*M. luteus* ATCC 10240	7.8	0.49	0.98	1.95	125	0.49	0.98	0.12	-
*B. subtilis* ATCC 6633	7.8	0.49	7.8	1.95	>1000	0.49	0.03	0.24	-
*B. cereus* ATCC 10876	7.8	7.8	7.8	1.95	>1000	3.9	0.12	0.98	-
Gram-negative bacteria									
*E. coli* ATCC 25922	>1000	>1000	>1000	>1000	>1000	1000	-	0.004	-
*P. mirabilis* ATCC 12453	>1000	>1000	>1000	>1000	>1000	>1000	-	0.03	-
*K. pneumoniae* ATCC 13883	>1000	>1000	>1000	>1000	>1000	>1000	-	0.06	-
*P. aeruginosa* ATCC 9027	>1000	>1000	>1000	>1000	>1000	1000	-	0.49	-
Yeasts									
*C. albicans* ATCC 102231	250	250	1000	15.6	>1000	1000	-	-	0.98
*C. parapsilosis* ATCC 22019	>1000	1000	1000	7.8	>1000	1000	-	-	1.95

**Table 3:** Cytotoxic activity of compounds **4**–**9**.

Compound	IC50-HaCaT [µg/mL]		SI IC50-HaCaT/MIC-MT
	MTT	SULF	SULF
**4**	>50	>50	>3.13
**5**	>50	>50	>25
**6**	9.49	>50	>6.25
**7**	>50	>50	>25
**8**	0.57	50	6.25
**9**	0.06	1.31	0.16

**Table 4:** Crystal data, data collection, and refinement details of compounds **4**–**6**.

	4	5	6
Crystal data			
Chemical formula	C_15.50_H_23.44_N_6_O_1.22_S	C_15_H_22_N_6_S	C_32_H_48.22_N_12_O_2.11_S_2_
*M*_r_	345.46	318.44	698.94
Crystal system	Triagonal	Monoclinic	Triagonal
Space group	$\;\;\;\;\;\;\;R\bar{3}:H$	*P*2_1_/*n*	$\;\;\;\;\;\;\;R\bar{3}:H$
*a*, *b*, *c* (Å)	23.2044 (3), 23.2044 (3), 17.2241 (2)	10.2776 (2), 11.0270 (2), 14.7731 (3)	23.47158 (15), 23.47158 (15), 34.2380 (3)
α, β, γ (°)	90, 90, 120	90, 107.077 (2), 90	90, 90, 120
*V* (Å^3^)	8031.7 (2)	1600.44 (6)	16335.2 (3)
*Z*	18	4	18
μ (mm^−1^)	1.75	1.84	1.72
Crystal size (mm)	0.47 × 0.19 × 0.14	0.74 × 0.40 × 0.06	0.6 × 0.4 × 0.2
*T*_min_, *T*_max_	0.587, 1.000	0.418, 1.000	0.740, 1.000
Data collection			
No. of measured, independent and observed [*I* > 2σ(*I*)] reflections	35,469, 3757, 3509	22,970, 3306, 3021	65,950, 7458, 6866
*R*_int_	0.037	0.056	0.078
(sin θ/λ)_max_ (Å^−1^)	0.637	0.636	0.637
Refinement			
*R*[*F*^2^ > 2σ(*F*^2^)], *wR*(*F*^2^), *S*	0.037, 0.094, 1.07	0.039, 0.108, 1.04	0.038, 0.100, 1.05
No. of reflections	3757	3306	7458
No. of parameters	227	208	501
Δ_max_, Δ_min_ (e Å^−3^)	0.57, −0.34	0.61, −0.37	0.33, −0.35

**Table 5:** Crystal data, data collection, and refinement details of compounds **7**–**9**.

	7	8	9
Crystal data			
Chemical formula	C_16_H_24_N_6_S	C_17_H_23_O_4_S	C_17_H_19_N_5_OS
*M*_r_	332.47	393.46	341.43
Crystal system	Triclinic	Orthorhombic	Monoclinic
Space group	$\;\;\;\;\;\;\;\;P\bar{1}$	*Pccn*	*P*2_1_/*c*
*a*, *b*, *c* (Å)	8.46333 (10), 15.03304 (15), 27.4526 (2)	38.0250 (5), 12.37094 (19), 7.92165 (12)	5.0283 (1), 22.5544 (3), 14.5985 (2)
α, β, γ (°)	98.1961 (8), 90.2187 (8), 100.2120 (9)	90, 90, 90	90, 97.451 (1), 90
*V* (Å^3^)	3400.82 (6)	3726.39 (9)	1641.64 (5)
*Z*	8	8	4
μ (mm^−1^)	1.76	1.85	1.87
Crystal size (mm)	0.45 × 0.22 × 0.18	0.54 × 0.25 × 0.20	0.5 × 0.2 × 0.1
*T*_min_, *T*_max_	0.338, 1.000	0.752, 1.000	0.526, 1.000
Data collection			
No. of measured, independent and observed [*I* > 2σ(*I*)] reflections	106,334, 14,102, 12,685	14,227, 3766, 3545	23,458, 3365, 3150
*R*_int_	0.079	0.023	0.058
(sin θ/λ)_max_ (Å^−1^)	0.636	0.636	0.637
Refinement			
*R*[*F*^2^ > 2σ(*F*^2^)], *wR*(*F*^2^), *S*	0.047, 0.131, 1.04	0.037, 0.091, 1.05	0.040, 0.111, 1.06
No. of reflections	14,102	3766	3365
No. of parameters	864	252	229
Δ_max_, Δ_min_ (e Å^−3^)	0.85, −0.54	0.49, −0.45	0.34, −0.39

**Table 6:** Hydrogen-bond geometry (Å, °) for **4**.

*D*—H···*A*	*D*—H	H···*A*	*D*···*A*	*D*—H···*A*
N4—H4A···S1 ^i^	0.87(2)	2.55(2)	3.3997(13)	167(2)
N4—H4B···S1 ^ii^	0.87(2)	2.68(2)	3.4601(13)	150(2)

Symmetry codes: (i) − *y* + 4/3, *x* – *y* + 2/3, *z* − 1/3; (ii) *y* − 1/3, − *x* + *y* + 1/3, − *z* + 4/3.

**Table 7:** Hydrogen-bond geometry (Å, °) for **5**.

*D*—H···*A*	*D*—H	H···*A*	*D*···*A*	*D*—H···*A*
N4—H4A···S1 ^i^	0.89(2)	2.45(2)	3.3295(13)	171.8(17)

Symmetry codes: (i)− *x* + 3/2, *y* − 1/2, − *z* + 1/2.

**Table 8:** Hydrogen-bond geometry (Å, °) for **6**.

*D*—H···*A*	*D*—H	H···*A*	*D*···*A*	*D*—H···*A*
N4A—H4AB···S1B	0.88(3)	2.48(3)	3.3022(16)	156(2)
N3B—H3B···S1B	0.83(2)	2.35(2)	2.8530(15)	120(2)
N4B—H4BA···S1A ^i^	0.89(3)	2.50(3)	3.3494(16)	161(2)
N4C—H4CB···S1D ^i^	0.90(3)	2.48(3)	3.3307(16)	157(2)
N4D—H4DA···S1C	0.88(3)	2.45(3)	3.2760(17)	156(2)

Symmetry codes: (i) *x* − 1, *y* − 1, *z*.

**Table 9:** Hydrogen-bond geometry (Å, °) for **7**.

*D*—H···*A*	*D*—H	H···*A*	*D*···*A*	*D*—H···*A*
N4—H4B···S1 ^i^	0.85(2)	2.48(2)	3.3061(15)	165(2)

Symmetry codes: (i)*x* + 1, − *y* + 1/2, *z* + 1/2.

**Table 10:** Hydrogen-bond geometry (Å, °) for **8**.

*D*—H···*A*	*D*—H	H···*A*	*D*···*A*	*D*—H···*A*
N4A—H4A···S1B	0.89(2)	2.49(2)	3.3485(13)	164.3(16)
N4A—H4B···S1A ^i^	0.84(2)	2.86(2)	3.5748(12)	143.9(16)
N3B—H3B···S1B	0.871(19)	2.349(18)	2.8316(12)	115.2(14)
N4B—H4C···S1A ^ii^	0.86(2)	2.59(2)	3.4157(13)	160.3(17)
N4B—H4D···S1B ^iii^	0.81(2)	2.58(2)	3.3085(13)	150.8(17)

Symmetry codes: (i)*x* − *y* + 2/3, *x* + 1/3, −*z* + 4/3; (ii) −*y* + 4/3, *x* – *y* + 2/3, *z*−1/3; (iii) *x* – *y* + 1, *x*, −*z* + 1.

**Table 11:** Hydrogen-bond geometry (Å, °) for **9**.

*D*—H···*A*	*D*—H	H···*A*	*D*···*A*	*D*—H···*A*
O1W—H1WA···O2W	0.76(2)	2.01(2)	2.7698(18)	174(2)
O1W—H1WB···S1 ^i^	0.86(2)	2.51(2)	3.3673(13)	177.1(18)
O2W—H2WA···S1 ^ii^	0.86(2)	2.45(2)	3.3097(12)	172.8(18)
O2W—H2WB···S1 ^iii^	0.86(2)	2.41(2)	3.2556(13)	169.1(18)
N4—H4A···O14Aa^, iv^	0.88	2.20	2.928 (4)	139.2
N4—H4A···O14Bb ^iv^	0.88	2.25	3.001(3)	143.8
N4—H4B···O1W	0.88	2.08	2.9164(17)	159.7

Symmetry codes: (i)*x*, −*y* + 3/2, *z* + 1/2; (ii) *x*, *y* + 1, *z*; (iii) *x*, −*y* + 3/2, *z*−1/2; (iv) −*x* + 1, *y* + 1/2, −*z* + 3/2.

## Data Availability

Data is contained within the article.

## Supplementary Materials

The following supporting information can be downloaded at: https://www.mdpi.com/article/10.3390/ma16010448/s1, Figure S1: ^1^H NMR spectrum (500 MHz, DMSO-*d_6_*) of compound **1**; Figure S2: ^13^C NMR spectrum (125 MHz, DMSO-*d*_6_) of compound **1**; Figure S3: ^1^H NMR spectrum (500 MHz, DMSO-*d_6_*) of compound **2**; Figure S4: 13C NMR spectrum (125 MHz, DMSO-*d_6_*) of compound **2**; Figure S5: ^1^H NMR spectrum (500 MHz, DMSO-*d_6_*) of compound **3**; Figure S6: ^13^C NMR spectrum (125 MHz, DMSO-*d*_6_) of compound **3**; Figure S7: ^1^H NMR spectrum (500 MHz, DMSO-*d_6_*) of compound **4**; Figure S8: ^13^C NMR spectrum (125 MHz, DMSO-*d*_6_) of compound **4**; Figure S9: ^1^H NMR spectrum (500 MHz, DMSO-*d_6_*) of compound **5**; Figure S10: ^13^C NMR spectrum (125 MHz, DMSO-*d*_6_) of compound **5**; Figure S11: ^1^H NMR spectrum (500 MHz, DMSO-*d_6_*) of compound **6**; Figure S12: ^13^C NMR spectrum (125 MHz, DMSO-*d*_6_) of compound **6**; Figure S13: 1H NMR spectrum (500 MHz, DMSO-*d_6_*) of compound **7**; Figure S14: ^13^C NMR spectrum (125 MHz, DMSO-*d*_6_) of compound **7**; Figure S15: ^1^H NMR spectrum (500 MHz, DMSO-*d_6_*) of compound **8**; Figure S16: ^13^C NMR spectrum (125 MHz, DMSO-*d*_6_) of compound **8**; Figure S17: ^1^H NMR spectrum (500 MHz, DMSO-*d_6_*) of compound **9**; Figure S18: ^13^C NMR spectrum (125 MHz, DMSO-*d*_6_) of compound **9**.
