# Supplementary material for: Synthesis and Structure–Activity Relationship of 2,6-Disubstituted Thiosemicarbazone Derivatives of Pyridine as Potential Antituberculosis Agents
Source: Materials (Basel). 2023 Jan 3;16(1):448. doi: 10.3390/ma16010448 (PMC9822072; doi:10.3390/ma16010448)
Supplement: Supplementary file 1 [file materials-16-00448-s001.zip › materials-2116636-supplementary.pdf]

Supplementary

Synthesis and structure–activity relationship of 2,6-disubstituted thiosemicarbazone derivatives of pyridine as potential antituberculosis agents

Dagmara Ziembicka *, Katarzyna Gobis *, Małgorzata Szczesio, Andrzej Olczak, Ewa Augustynowicz-Kopeć, Agnieszka Głogowska, Izabela Korona-Głowniak and Krzysztof Bojanowski

Figures S1–S18 show ^{1}H NMR and ^{13}C NMR spectra of compounds **1–9**.

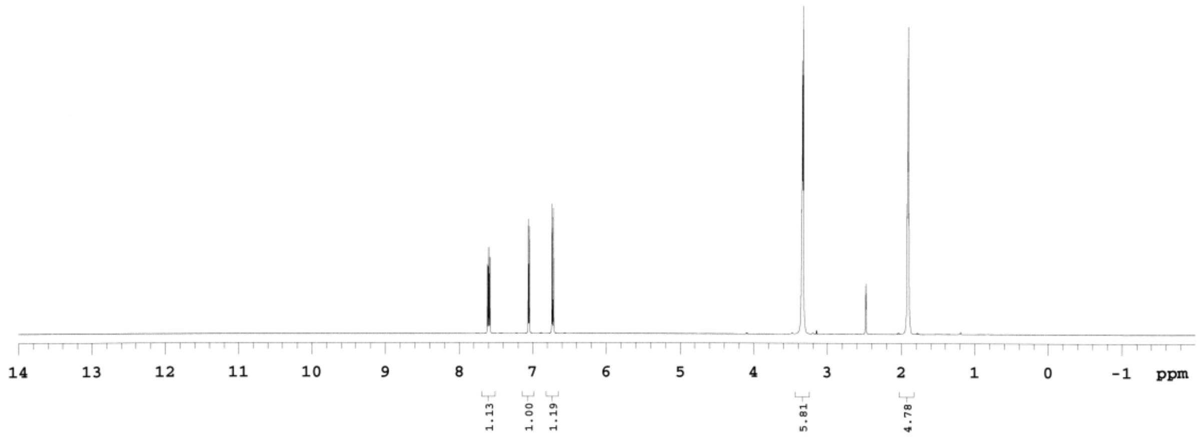

Figure S1. ^{1}H NMR spectrum (500 MHz, DMSO-d_6) of compound **1**.

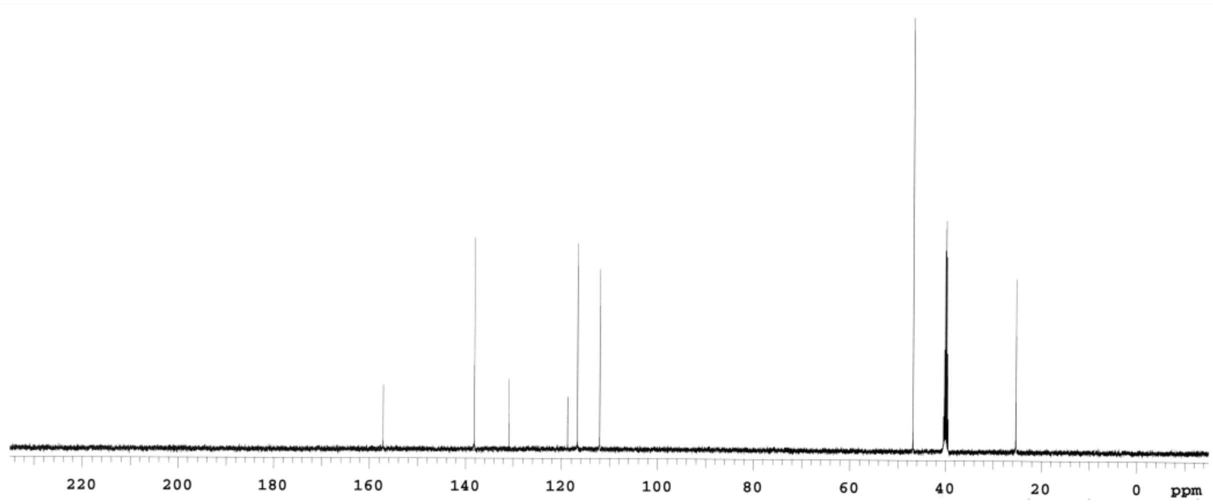

Figure S2. ^{13}C NMR spectrum (125 MHz, DMSO-d_6) of compound **1**.

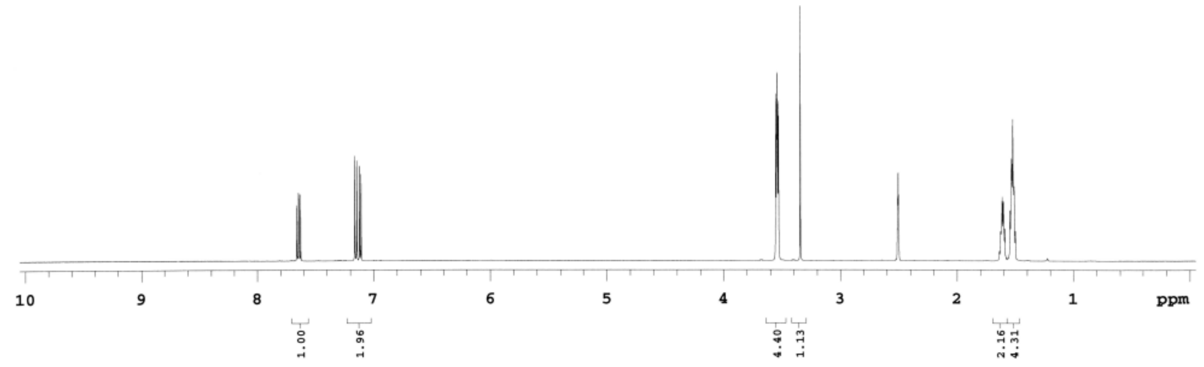

Figure S3. ^{1}H NMR spectrum (500 MHz, DMSO-d_6) of compound **2**.

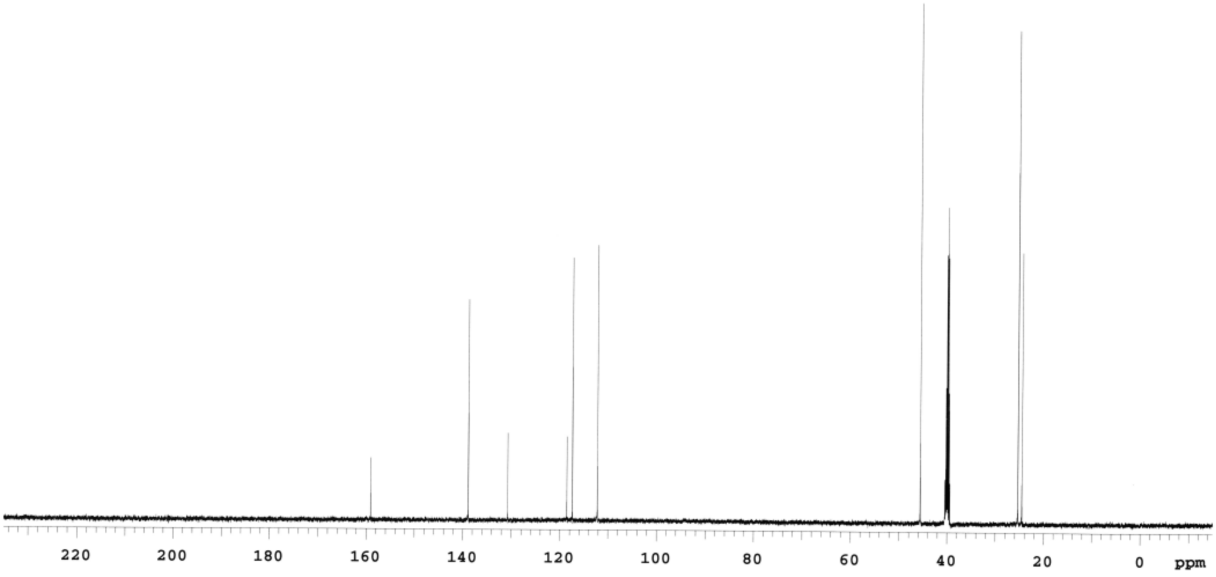

Figure S4. ^{13}C NMR spectrum (125 MHz, DMSO-d_6) of compound **2**.

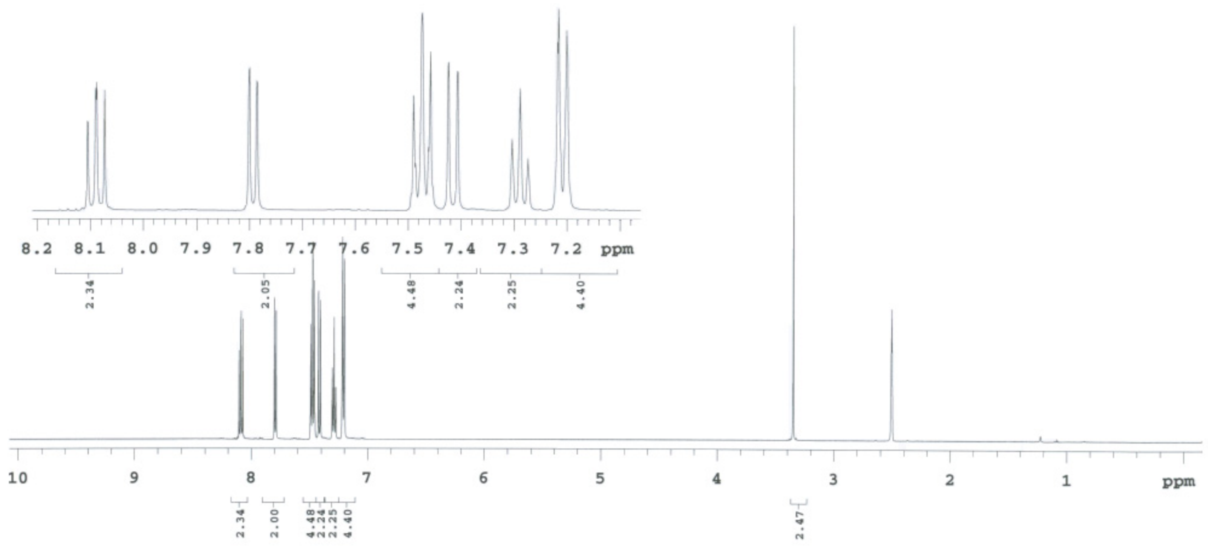

Figure S5. ^{1}H NMR spectrum (500 MHz, DMSO-d_6) of compound **3**.

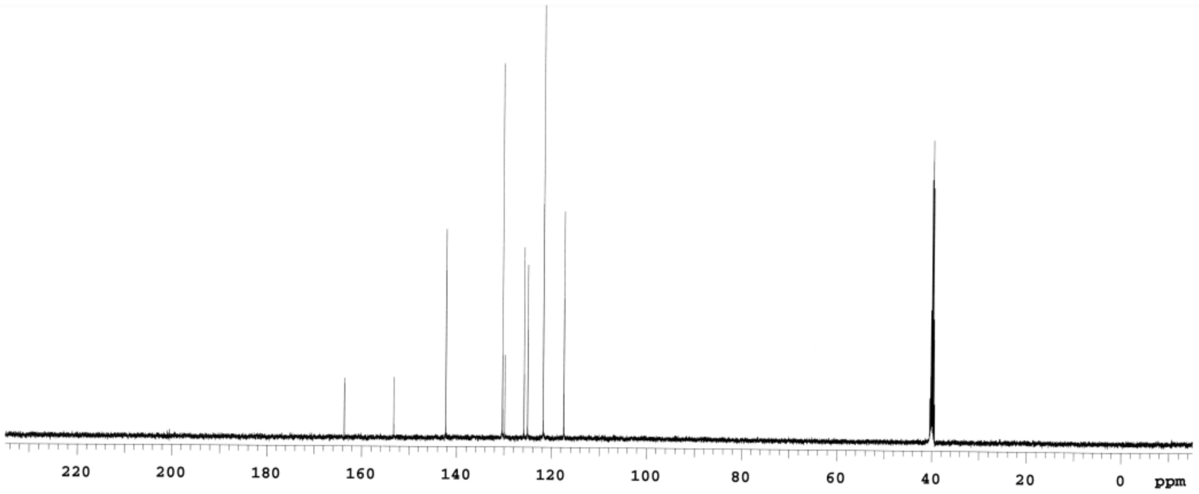

Figure S6. ^{13}C NMR spectrum (125 MHz, DMSO-d_6) of compound **3**.

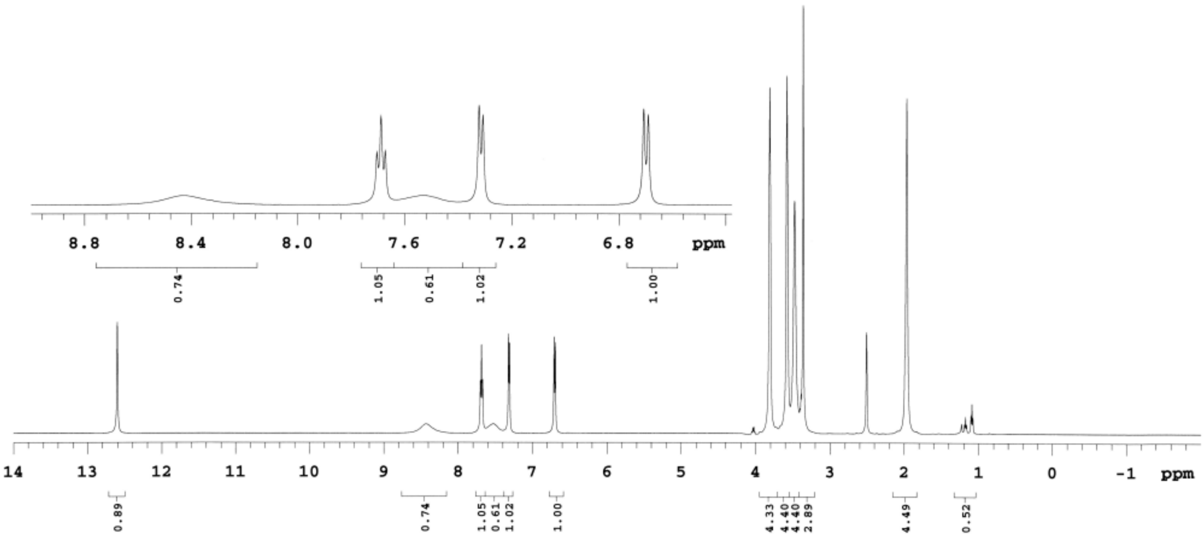

Figure S7. ^{1}H NMR spectrum (500 MHz, DMSO-d_6) of compound **4**.

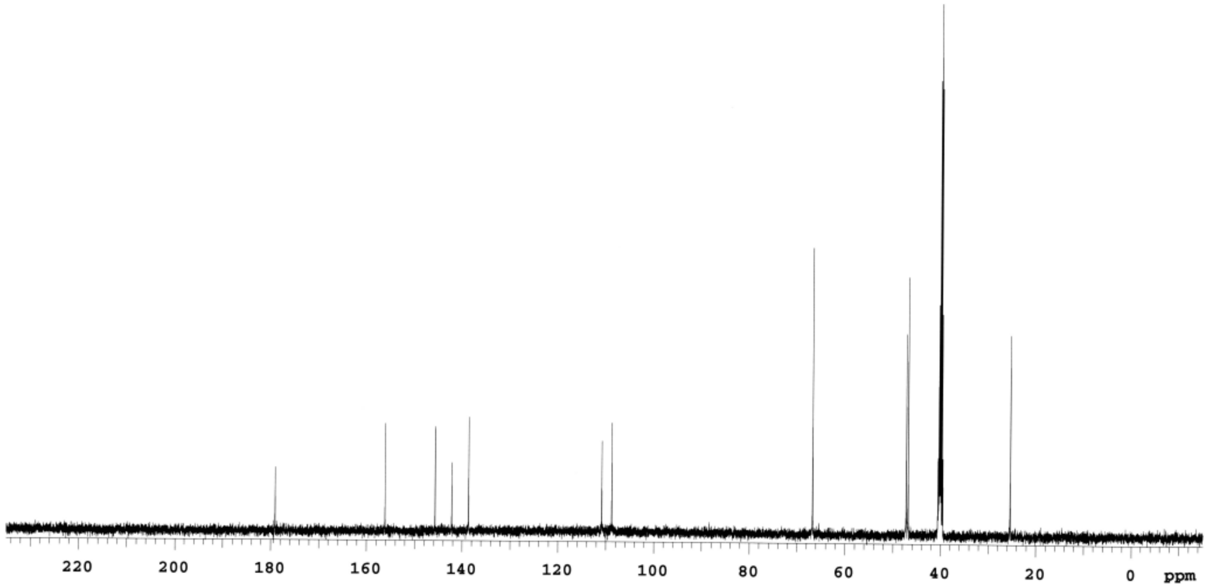

Figure S8. ^{13}C NMR spectrum (125 MHz, DMSO-d_6) of compound **4**.

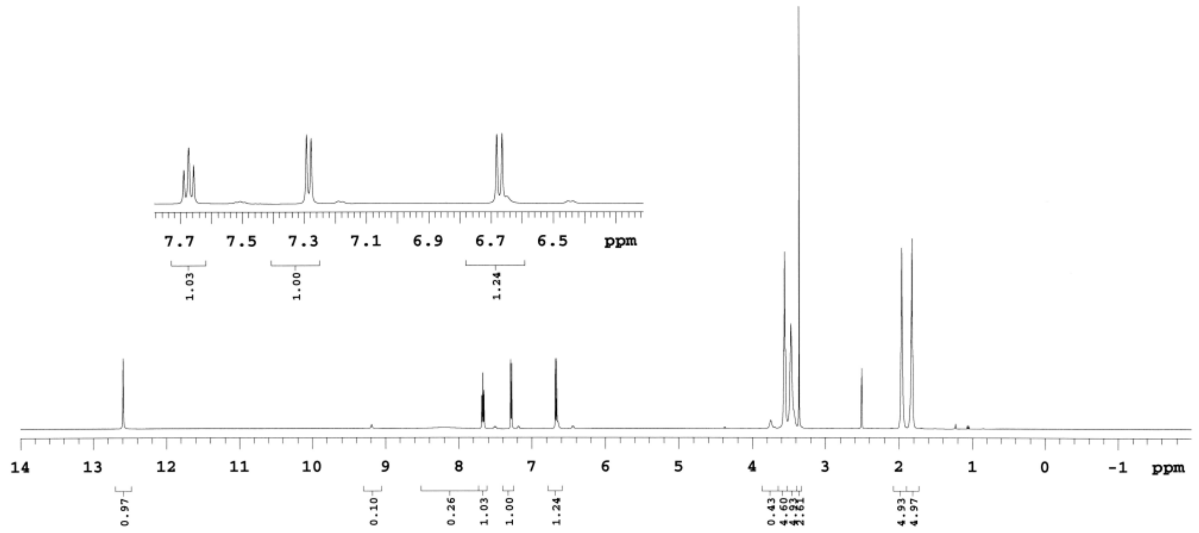

Figure S9. ^{1}H NMR spectrum (500 MHz, DMSO-d_6) of compound **5**.

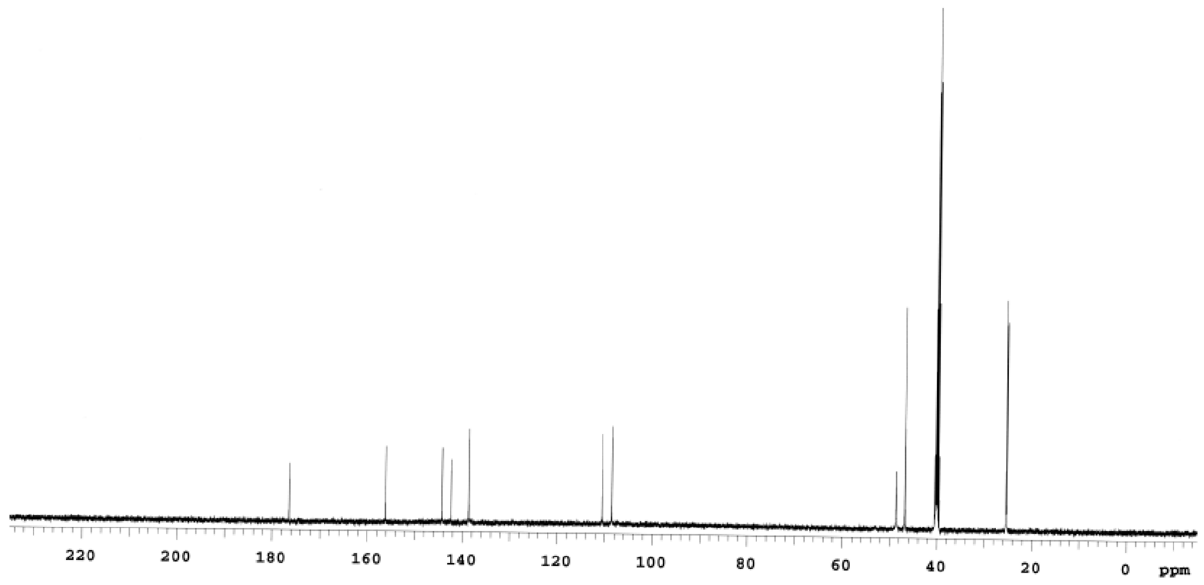

Figure S10. ^{13}C NMR spectrum (125 MHz, DMSO-d_6) of compound **5**.

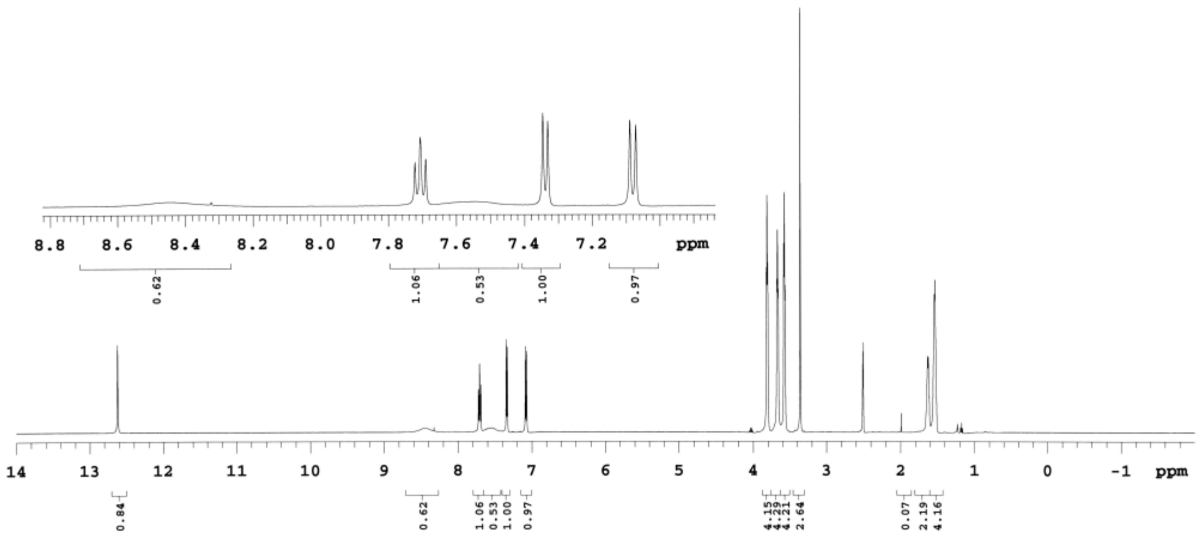

Figure S11. ^{1}H NMR spectrum (500 MHz, DMSO-d_6) of compound **6**.

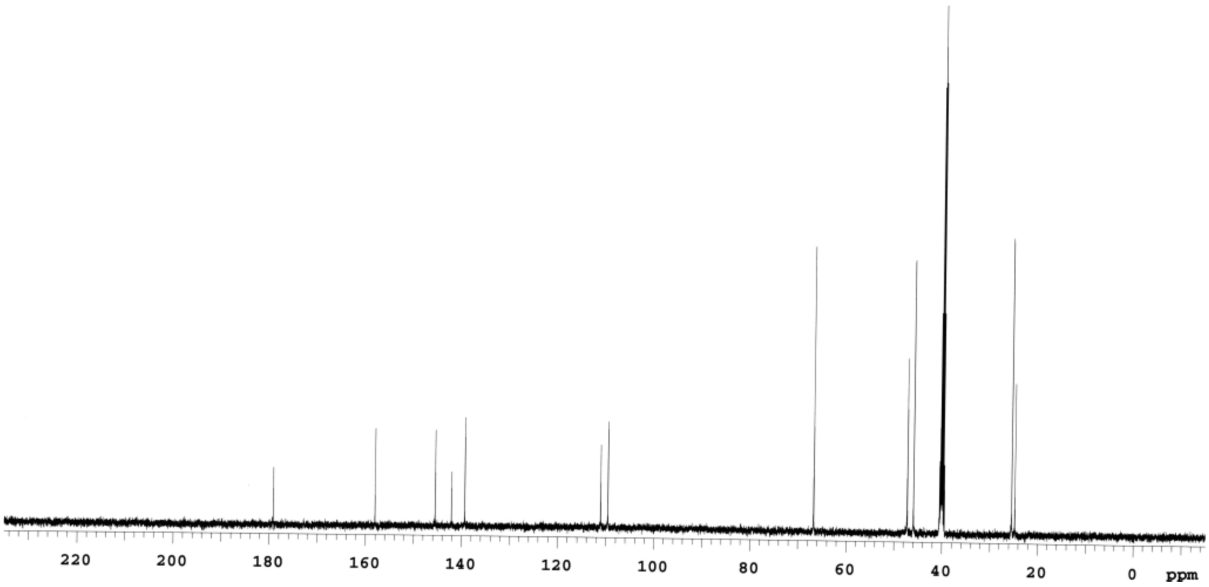

Figure S12. ^{13}C NMR spectrum (125 MHz, DMSO-d_6) of compound **6**.

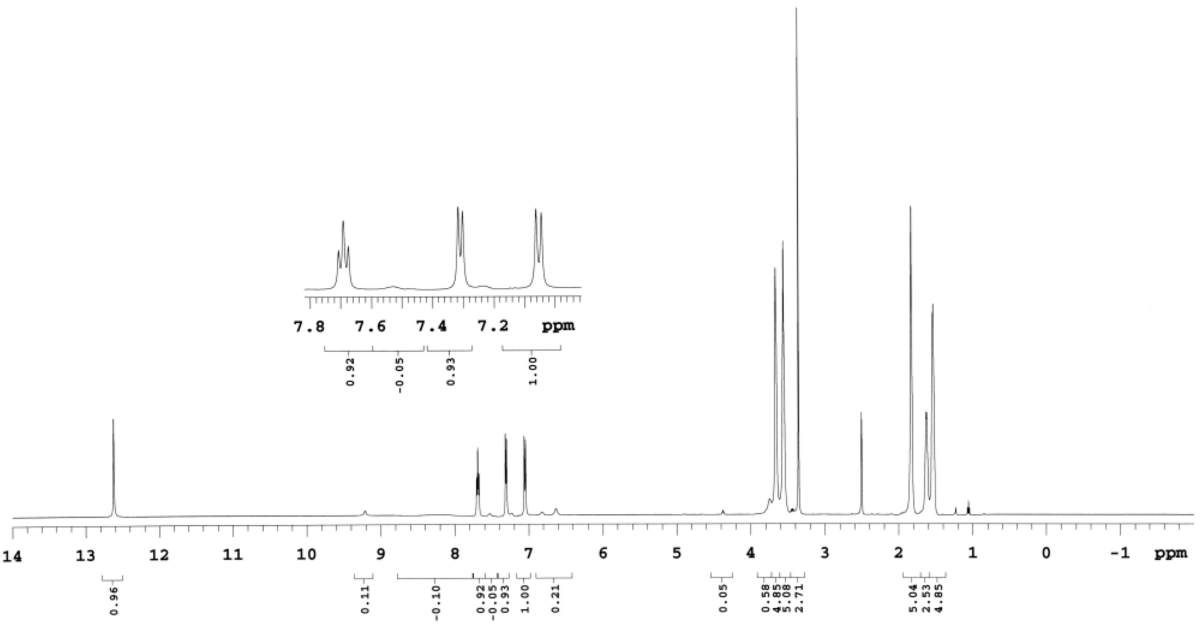

Figure S13. ^{1}H NMR spectrum (500 MHz, DMSO-d_6) of compound **7**.

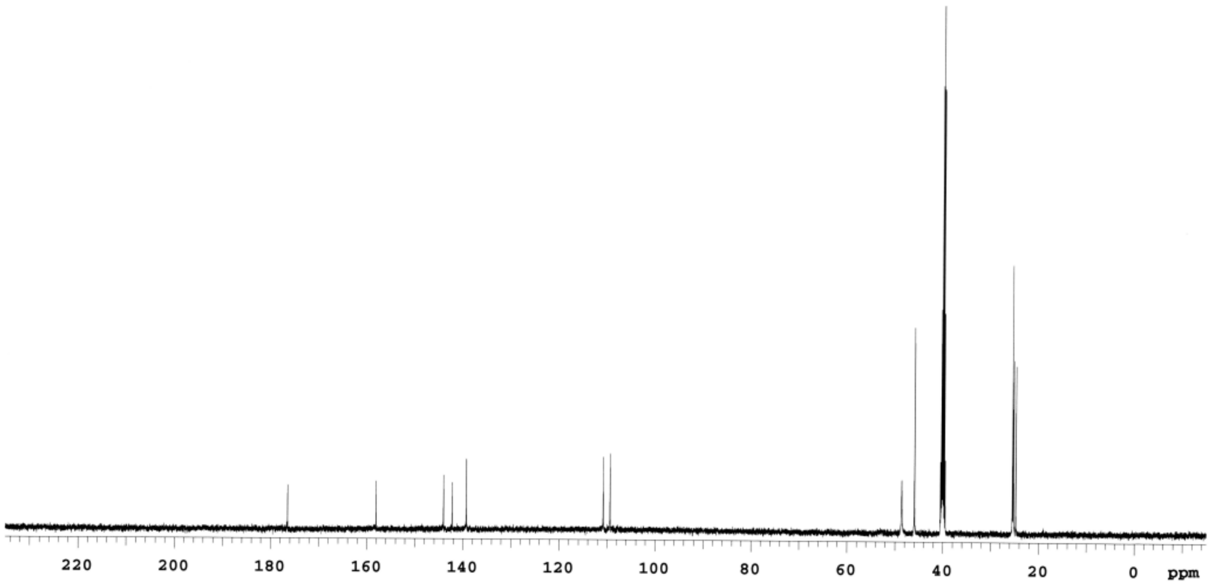

Figure S14. ^{13}C NMR spectrum (125 MHz, DMSO-d_6) of compound **7**.

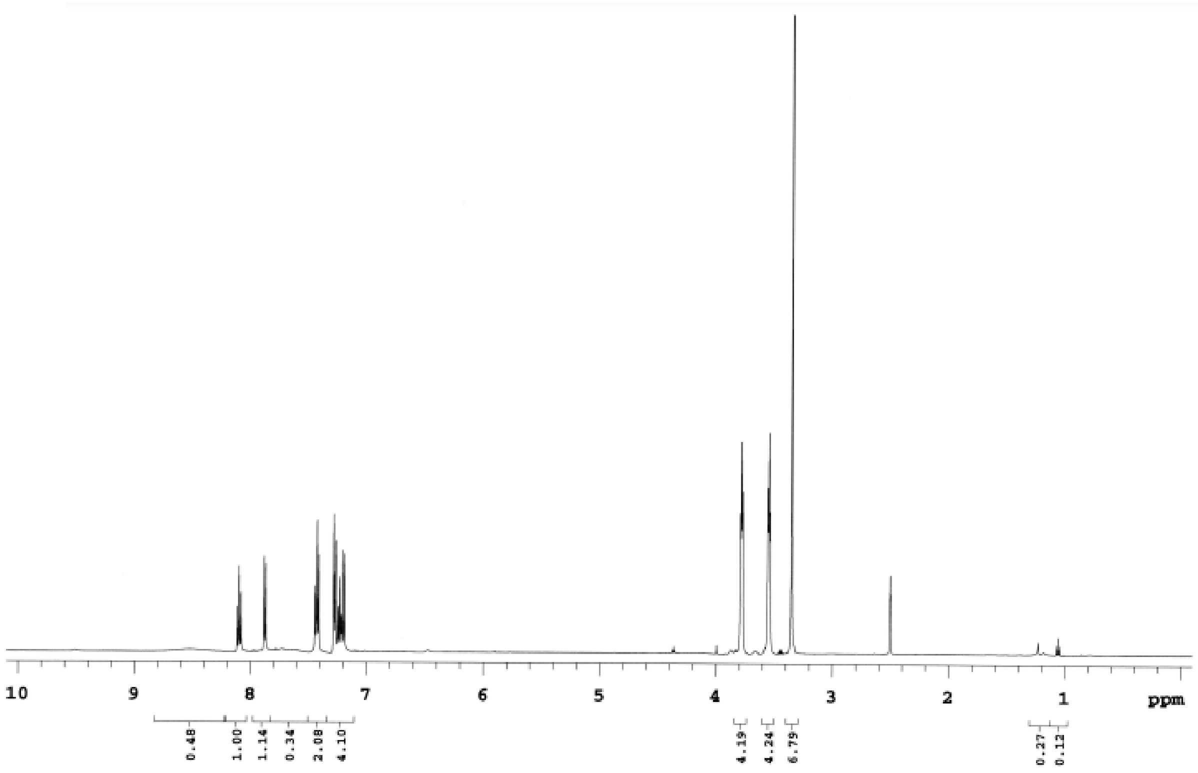

Figure S15. ^{1}H NMR spectrum (500 MHz, DMSO-d_6) of compound **8**.

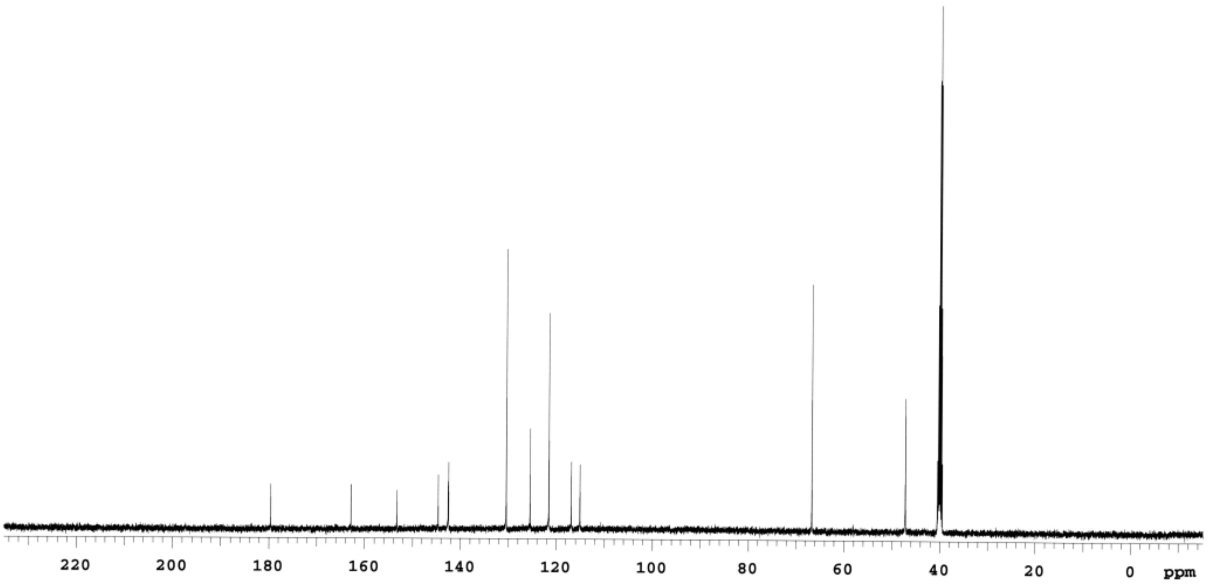

Figure S16. ^{13}C NMR spectrum (125 MHz, DMSO-d_6) of compound **8**.

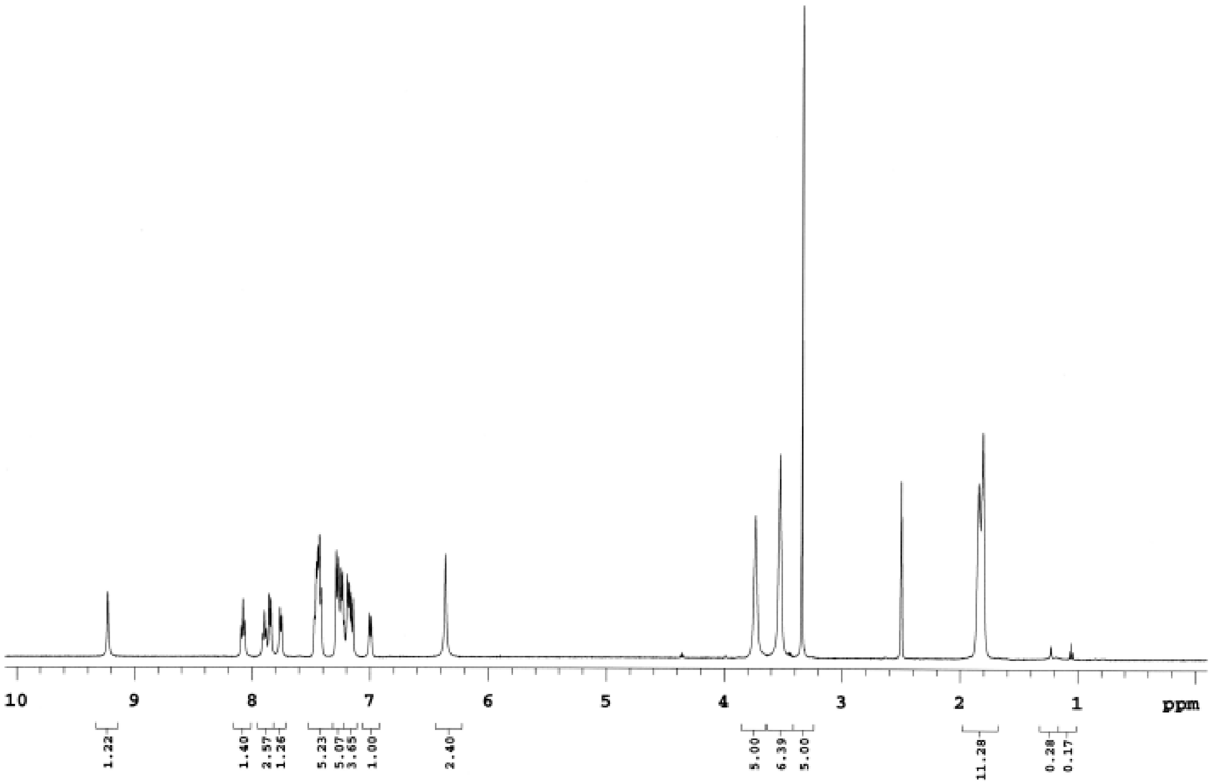

Figure S17. ^{1}H NMR spectrum (500 MHz, DMSO-d_6) of compound **9**.

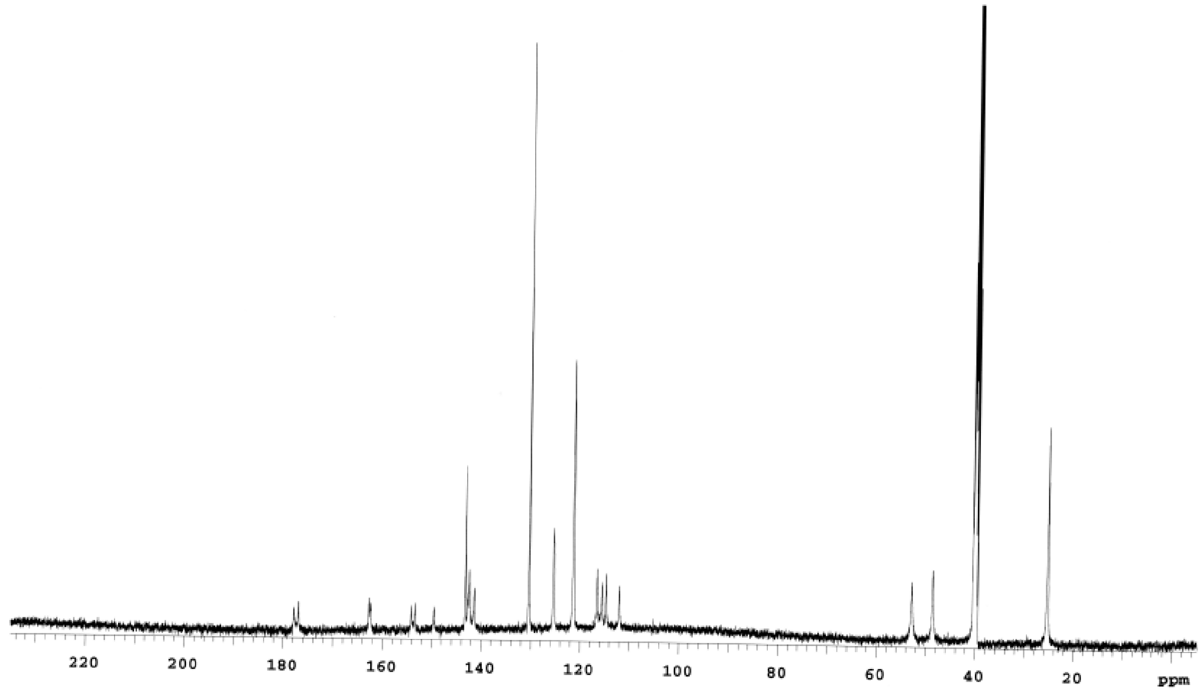

Figure S18. ^{13}C NMR spectrum (125 MHz, DMSO-d_6) of compound **9**.
